# Mechanisms of a Cyclobutane-Fused Lactone Hydrolysis in Alkaline and Acidic Conditions

**DOI:** 10.3390/molecules26123519

**Published:** 2021-06-09

**Authors:** Zhangxia Wang, Haibo Ma

**Affiliations:** Jiangsu Key Laboratory of Vehicle Emissions Control, School of Chemistry and Chemical Engineering, Nanjing University, Nanjing 210023, China; wangzhangxia@smail.nju.edu.cn

**Keywords:** hydrolysis mechanisms, cyclobutane-fused lactone, DFT, solvation model

## Abstract

Searching for functional polyesters with stability and degradability is important due to their potential applications in biomedical supplies, biomass fuel, and environmental protection. Recently, a cyclobutane-fused lactone (CBL) polymer was experimentally found to have superior stability and controllable degradability through hydrolysis reactions after activation by mechanical force. In order to provide a theoretical basis for developing new functional degradable polyesters, in this work, we performed a detailed quantum chemical study of the alkaline and acidic hydrolysis of CBL using dispersion-corrected density functional theory (DFT-D3) and mixed implicit/explicit solvent models. Various possible hydrolysis mechanisms were found: B_AC_2 and B_AL_2 in the alkaline condition and A_AC_2, A_AL_2, and A_AL_1 in the acidic condition. Our calculations indicated that CBL favors the B_AC_2 and A_AC_2 mechanisms in alkaline and acidic conditions, respectively. In addition, we found that incorporating explicit water solvent molecules is highly necessary because of their strong hydrogen-bonding with reactant/intermediate/product molecules.

## 1. Introduction

**Scheme 1:**
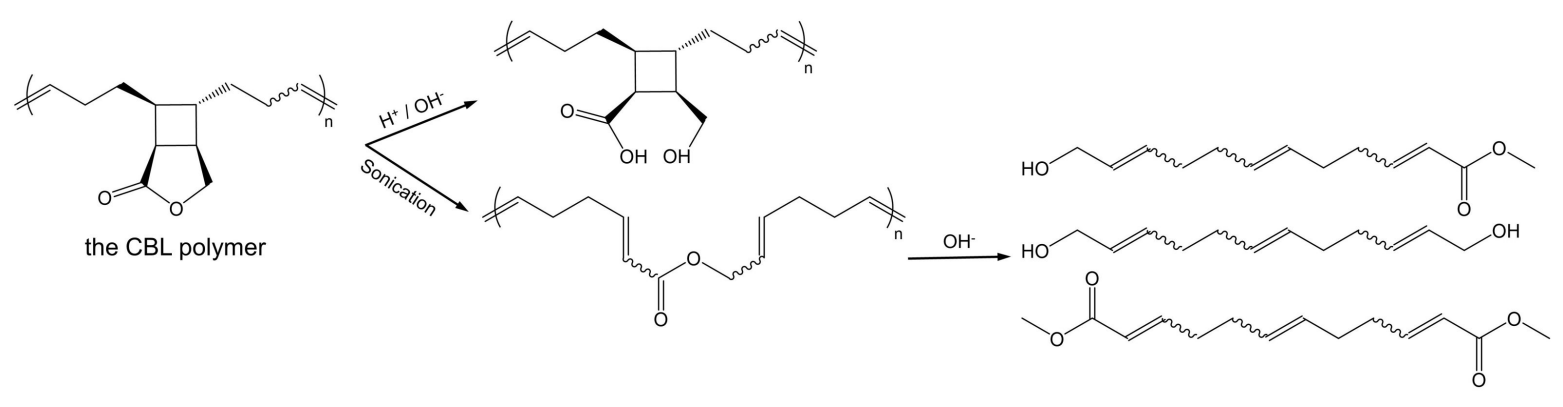
The cyclobutane-fused lactone (CBL) polymer (i) hydrolysis in alkaline or acidic conditions and (ii) mechanochemical activation after sonication.

Recently, a cyclobutane-fused lactone (CBL) polymer was proposed and demonstrated to have superior stability and controllable degradability [1]. Cyclobutane shows a perfect stability to keep the polymer’s backbone when the ester groups are hydrolyzed under alkaline and acidic conditions. When degradation is needed, the CBL polymer can be degraded through hydrolysis reactions after activation by mechanical force (Scheme 1). Hopefully, functional materials with degradability and stability will be developed based on the special chemical structure of CBL, especially in biomedical applications, but there have been no relevant research reports so far. At the same time, theoretical research on the hydrolysis mechanism of CBL is lacking. In order to provide the basis for the application of CBL polymer materials, we need to unravel its hydrolysis mechanisms.

The hydrolysis mechanisms of CBL are complex, and there are different reaction routes in different chemical environments. Solvent molecules also have a great influence on the hydrolysis reaction, because water molecules play more than one role in hydrolysis: acting as solvent, proton-carrier, and catalyst. Moreover, there are strong hydrogen-bonding interactions between water molecules and reactant/intermediate/product molecules that cannot be ignored in the study of the reaction mechanism [2]. Therefore, it is necessary to study the functions of explicit solvent molecules and the influence of various numbers of water molecules in detail.

Lactone hydrolysis has been widely studied in organic chemical experiments and biological systems. A comprehensive theoretical calculation work by Gómez-Bombarelli et al., summarized the features of the various possible hydrolysis mechanisms of two linear esters and eight lactones [3,4]. In this work, they directly adopted a model of one ester molecule including six water molecules. Though employing explicit solvation models is reliable for studying reaction mechanisms [5,6,7,8,9,10,11,12,13], they ignored the effect of a varying number of water molecules on the hydrolysis processes. CBL is similar to lactones in structure, so it features all the possible hydrolysis mechanisms of lactones. Compared with γ-butyrolactone, CBL has one more cyclobutane in its chemical structure, which may affect its hydrolysis mechanism.

In this work, the hydrolysis mechanisms of CBL were investigated by density functional theory (DFT) calculations with explicit solvation models. The classification of ester hydrolysis mechanisms proposed by Ingold [14] was adopted: the capital letters A and B stand for acid- and base-catalyzed, respectively; the subscripts _AC_ and _AL_ stand for acyl– or alkyl–oxygen cleavage, respectively; and the last numbers 1 and 2 stand for the uni- or bimolecular pathway, respectively. A detailed comparison of CBL hydrolysis with pure implicit solvent and mixed implicit/explicit solvent models was discussed. The calculation results showed that CBL follows B_AC_2 (activation free energy, Δ*G^≠^* = 15.4 kcal/mol) and A_AC_2 (Δ*G^≠^* = 24.2 kcal/mol) mechanisms in alkaline and acidic conditions, respectively. B_AC_2 (base catalyzed nucleophilic addition followed by hydrolysis) and A_AC_2 (acid catalyzed hydrolysis) mechanisms are the two most important pathways in ester hydrolysis in practice, and both of them proceed via a tetrahedral intermediate [15]. A_AL_1 has been rarely reported, and we also found that it is unlikely to occur because of a high activation free energy of 37.1 kcal/mol. At the same time, our calculations indicated that using a purely implicit solvation model will underestimate activation free energies by ~4 kcal/mol in B_AC_2 when compared with incorporating additional explicit water solvent molecules, and the activation free energies will not change much when including more than two explicit water molecules.

## 2. Computational Details

Quantum chemical calculations based on dispersion-corrected DFT [16] were performed using Gaussian 16 Rev. A.03. [17] The geometries of reactants (R), transition states (TS), intermediates (Int), and products (P) were optimized with no constraints at the B3LYP-D3/6-311+G(d) level [18] for studying the basic hydrolysis, whereas the 6-311++G(d,p) basis set was used to obtain the stationary points under the acidic condition. Solvent model density (SMD) [19] was selected to account for the contribution of solvation free energy to the total energy. The B3LYP-D3/6-311+G(d) theory level has been used for similar systems like the alkaline hydrolysis reaction of γ-butyrolactone [3,4], and the difference in activation free energy was found to be 0.1 kcal/mol at B3LYP-D3/6-311++G(2df,pd) level (Table S1). We also tested the M06-2X functional, and Tables S1 and S2 in the Supplementary Materials show that using B3LYP-D3 could get results that were consistent with the experiment for the alkaline hydrolysis reaction of γ-butyrolactone. Free energies were obtained relative to the R of each pathway, and the lowest energy R was chosen as a reference.

A common approach for including specific solute–solvent interactions is the use of a combination of dielectric continuum and explicit solvent molecules [11], an approach that has been called discrete-continuum [20], cluster-continuum [21], or implicit/explicit solvation [22]. Using this approach is effective to describe chemical reactions in aqueous solutions—particularly the hydrolysis reactions of amides [23,24,25,26,27,28,29], phosphates [7,30,31,32,33,34], and esters [3,4,5,6,35,36,37,38]. Therefore, we used the implicit/explicit solvation model to consider solvent effects for the CBL hydrolysis reaction. Stationary points (R/TS/Int/P) were optimized with the addition of 0–6 water molecules to the system. The water molecules were added one-by-one by hand in the initial structure, thus ensuring that each water molecule was not isolated. Additional water molecules of each transition state were added near the nucleophile group, the leaving group, and the CBL oxygen atoms. Another situation where water molecules could form hydrogen bonds with each other to stabilize the structures was also considered. In short, the additional water molecules should form more than one hydrogen bond with adjacent molecules. All the structures were reoptimized, and thermochemical values were computed at a room temperature of 298 K using uncorrected frequencies. The intrinsic reaction coordinate (IRC) paths [39] connecting the reactants and products were calculated to ensure that the transition states were reliable. The Cartesian coordinates and SCF energies of all optimized stationary points are provided in the Supplementary Materials. Because the CBL hydrolysis reactions are reversible and multiple, the Gibbs energy of activation Δ*G^≠^* derived from experiment [18,40] was given by Equation (1):

(1) $$\Delta G^{\neq }=\Delta G_{TS1}+\Delta G_{TS2}-\Delta G_{Int}+RTln\left(e^{-\frac{\Delta G_{TS2}-\Delta G_{Int}}{RT}}+e^{-\frac{\Delta G_{TS1}-\Delta G_{Int}}{RT}}\right)$$

## 3. Results and Discussion

### 3.1. Alkaline Hydrolysis

**Scheme 2:**
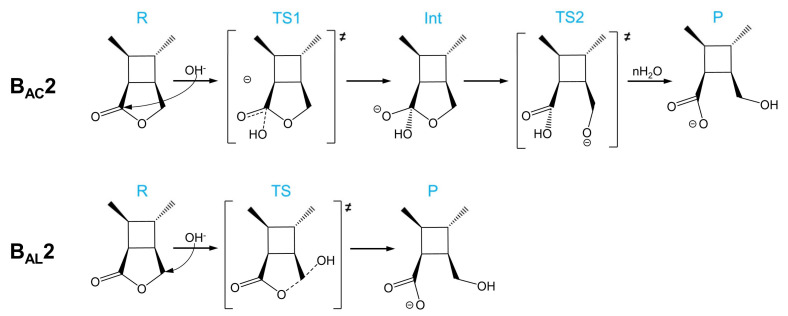
B_AC_2 and B_AL_2 mechanisms of the alkaline hydrolysis of CBL.

As shown in Scheme 2, there are two base-catalyzed mechanisms of CBL hydrolysis: B_AC_2 and B_AL_2. The B_AC_2 mechanism can be divided into two steps: the addition step and the elimination step. The nucleophile OH^−^ attacks the carbonyl carbon to form a tetrahedral intermediate (Int), and then the acyl–oxygen bond breaks before a process of intermolecular proton transfer (H_2_O attacks the ether O). In the B_AL_2 mechanism, the OH^−^ attacks the alkoxyl carbon from the opposite direction and the alkyl–oxygen bond is damaged at the same time.

#### 3.1.1. The B_AC_2 Mechanism: Deeper Insight by Explicit Solvent Models

In recent years, it has become popular to model ester hydrolysis and other reactions by adopting mixed implicit/explicit solvent models, which are especially useful in reactions involving ionic species [7]. To investigate whether the explicit solvent molecules had a substantial impact on the energies and geometries of the optimized stationary points, we performed geometrical optimizations for the B_AC_2 mechanism pathway of CBL with 0–6 water molecules using B3LYP-D3/6-311+G(d) in SMD. It is difficult to calculate all the microsolvated isomers and conformers for R, TS1, Int, and TS2 of CBL, especially when more and more water molecules are added to the system. Our strategy was to choose the configuration with the lowest energy for the following discussion. Here, TS1 was the transition state of the addition step, and TS2 was the transition state of the elimination step. Because the relative free energies of TS1, Int, and TS2 were based on R, we chose the R conformations carefully. We manually placed the water molecules to make the system form as many as hydrogen bonds as possible, and in this way, we chose the lowest energy conformation. Another method for choosing the lowest energy conformation was to locate the possible reactant complex following the IRC and then reoptimize it. Finally, compared the values obtained by the two ways and selected the one with the lowest energy. We found that the water molecules and OH^−^ gathered together to form a cluster.

**Figure 1:**
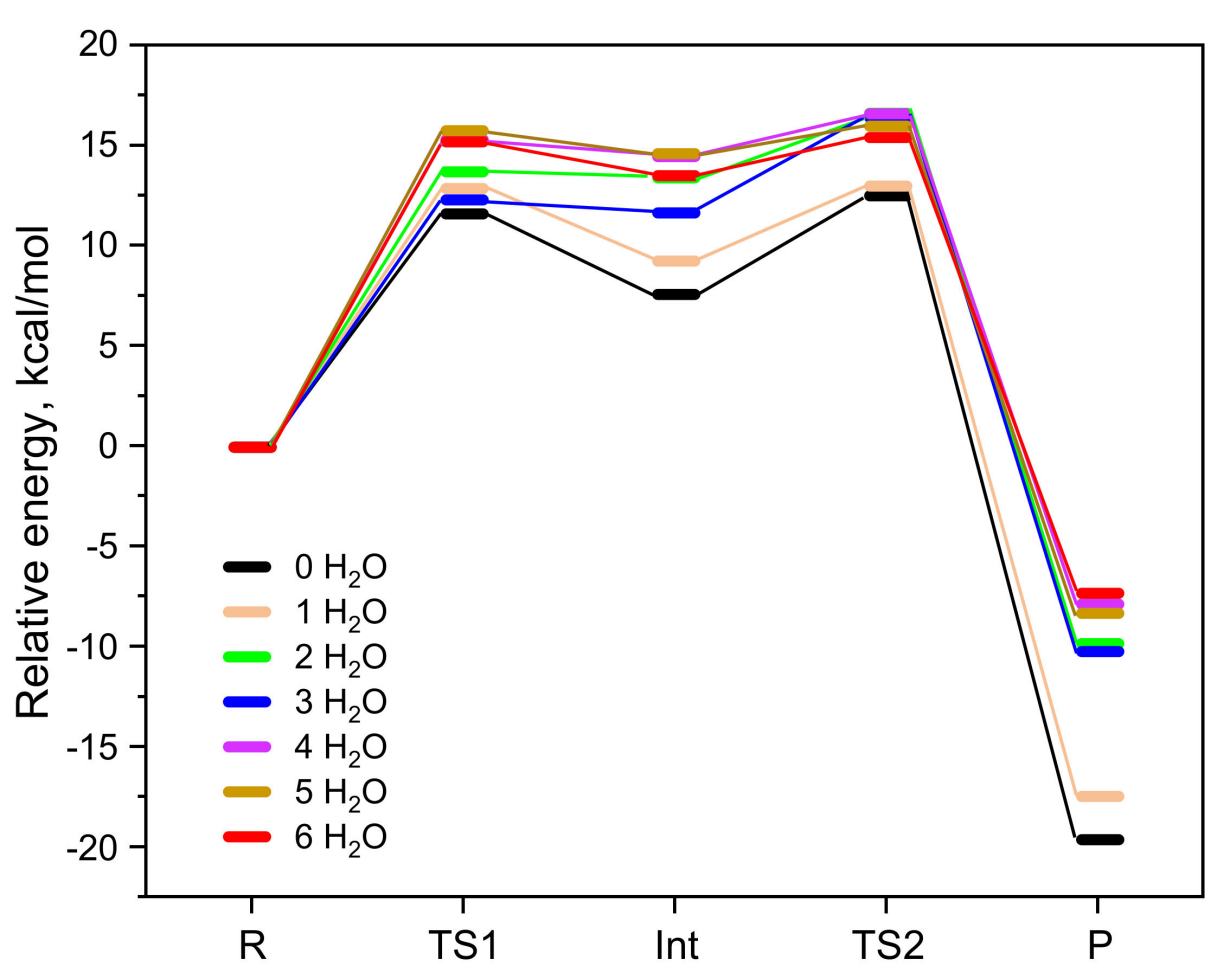
Comparison of the results of the B_AC_2 reaction path of CBL without microsolvation and with 1–6 H_2_O molecules. Free energies of TS1, Int, TS2, and P are relative to the R of each pathway.

**Figure 2:**
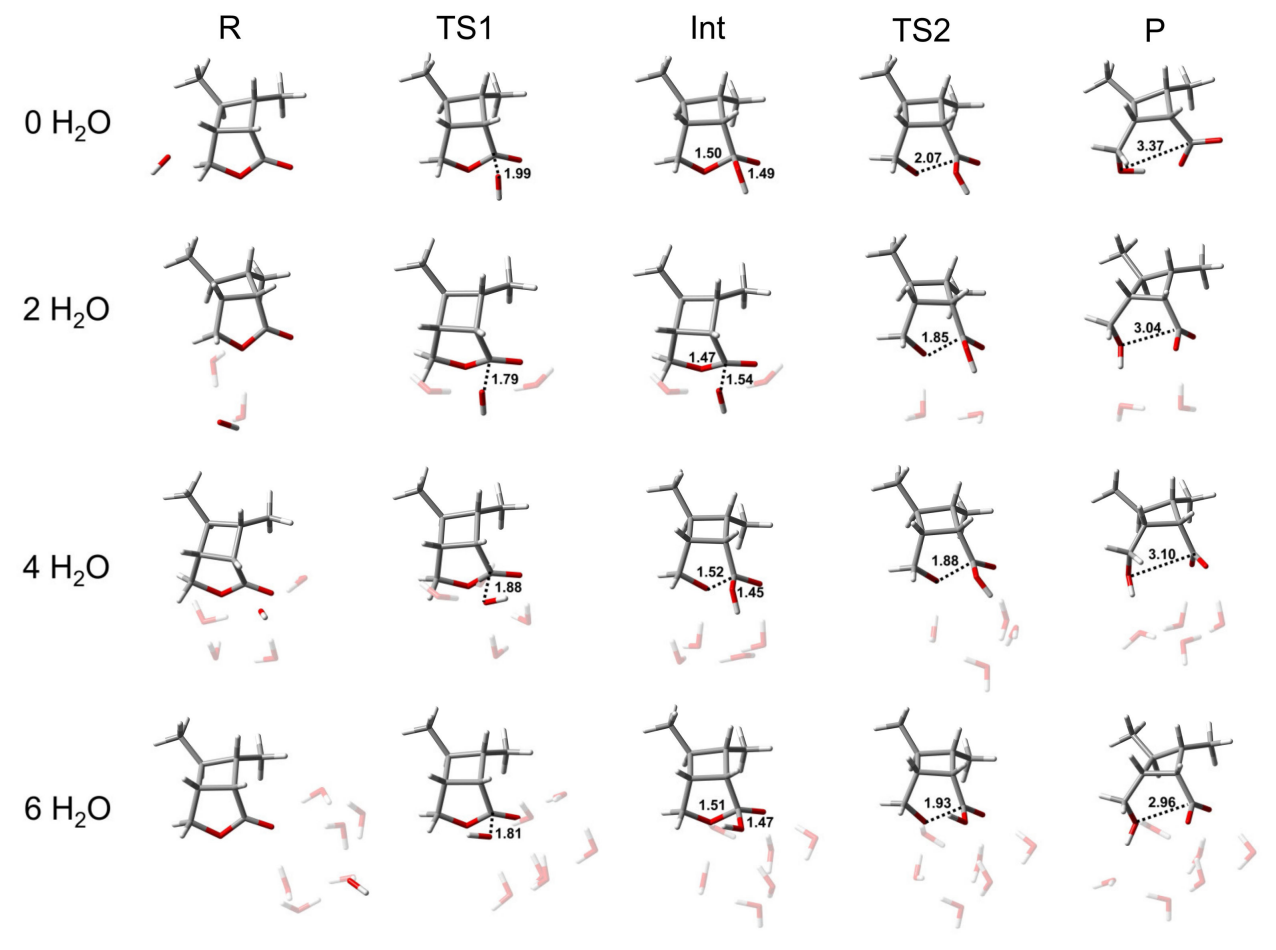
Representative structures of R, TS1, Int, TS2, and P of the B_AC_2 reaction path of CBL, with varying numbers of H_2_O molecules. The unit of distance is Å. The coordinates of all optimized stationary points are provided in the Supplementary Materials.

The results of the B_AC_2 reaction path of CBL with varying numbers of water molecules are shown in Figure 1 and Table 1. From these data, it can be seen that the relative free energies of TS1, Int, and TS2 were all the smallest when no water molecules were included, resulting in the smallest activation free energy (Δ*G^≠^* = 12.6 kcal/mol), followed by the presence of only one water molecule (Δ*G^≠^* = 13.3 kcal/mol). When the number of water molecules added to the model was greater than one, the relative free energies of the stationary points were almost the same and the calculated activation free energies were in the range of 15.7–16.6 kcal/mol. From this, we got an average activation free energy of 15.4 ± 2.8 kcal/mol. This indicated that incorporating explicit water solvent molecules is highly necessary because of their strong hydrogen-bonding with reactant/intermediate/product molecules. For example, water molecules formed hydrogen bonds with OH^−^ and the alkoxyl oxygen and carbonyl oxygen of CBL (Figure 2). After placing six explicit water molecules, the Δ*G^≠^* of the B_AC_2 reaction path converged to average value. Therefore, we did not add further water molecules.

While the energetics of the B_AC_2 reaction did not seem to be remarkably affected by the inclusion of the water molecules (>2H_2_O), the explicit solvent effect for the transition state geometries was significant. Adding an increasing number of explicit water molecules to the B_AC_2 reaction tightened the transition state by up to 0.18 or 0.25 Å in the C-O_nuc_ or C-O_lg_ distance, respectively (In Figure 2 and Table S3, C-O_nuc_ or C-O_lg_ denotes the bond lengths to the nucleophile or leaving group). This illustrates that TS1 and TS2 are better solvated by adding explicit water molecules to introduce hydrogen-bonding interactions. Similar changes also appeared in the product. The C-O_lg_ distance of the product reduce from 3.37 Å (no water molecules are present) to 2.96 Å (six water molecules are present), i.e., a reduction of around 12%. For the intermediate, the bond lengths of C-O_nuc_ and C-O_lg_ did not change.

In our calculations, we found that when there was no or only one water molecule in the model, we got the transition state of intramolecular proton transfer (as shown in Figure S1 in the Supplementary Materials). The relative free energy of the proton transfer step was 13.0 kcal/mol when no water molecules were present and 17.3 kcal/mol when one water molecule was present, and both were higher than the energy barriers of the nucleophilic addition and the leaving group departure. Therefore, the intramolecular proton transfer was unfavorable in terms of energy within this approximation. Furthermore, the intramolecular proton transfer never happened in alkaline conditions. In fact, the proton may transfer through multiple water molecules to achieve the deprotonation of the nucleophile and protonation of the leaving group. However, it would have been too computationally consuming to explore the full proton transfer network. Additionally considering the proton transfer step is usually not rate-limiting in the base-catalyzed hydrolysis of lactones [3] and phthalate esters [7,38], in this work, we did not investigate the intermolecular proton transfer in detail, but the tendency of protonation of the leaving group was observed in the IRC results (see Figure S2). This showed that the intermolecular proton transfer was spontaneous. Meanwhile, a relaxed potential energy surface scan was performed to estimate the deprotonation and protonation barrier of CBL + 6H_2_O + OH^−^ (Figure S3). We fixed the positions of the alkyl–oxygen and the hydrogen atom of H_2_O. The initial length of O···HOH was 1.78 Å, and then scan steps of 0.02 Å were carried out (0.98–1.78 Å). It can be seen from the results that the deprotonation of the nucleophile and protonation of the leaving group happened at the same time and that the electronic energy barrier (Δ*E*) was about 10 kcal/mol.

Cyclobutane had little effect on structures of the transition state along the B_AC_2 reaction pathway of CBL compared to γ-butyrolactone. The only difference was that the structure of the product obtained by hydrolysis through the B_AC_2 pathway remained unchanged (the initial “lactone group” remained cyclic after hydrolysis; see Figure 2), while the product of γ-butyrolactone without cyclobutane was linear [3,4]. This suggests that the reversible reaction of CBL hydrolysis is structurally advantageous compared to γ-butyrolactone, and this feature is expected to develop CBL polymers that can achieve industrial recyclability. Table S4 presents the dihedral angles (C-C-C-C) to the cyclobutane at each stationary point. No matter how many water molecules were added to the model, the cyclobutane in the products was twisted and the dihedral angles increased by 8°.

#### 3.1.2. The B_AL_2 Mechanism

**Figure 3:**
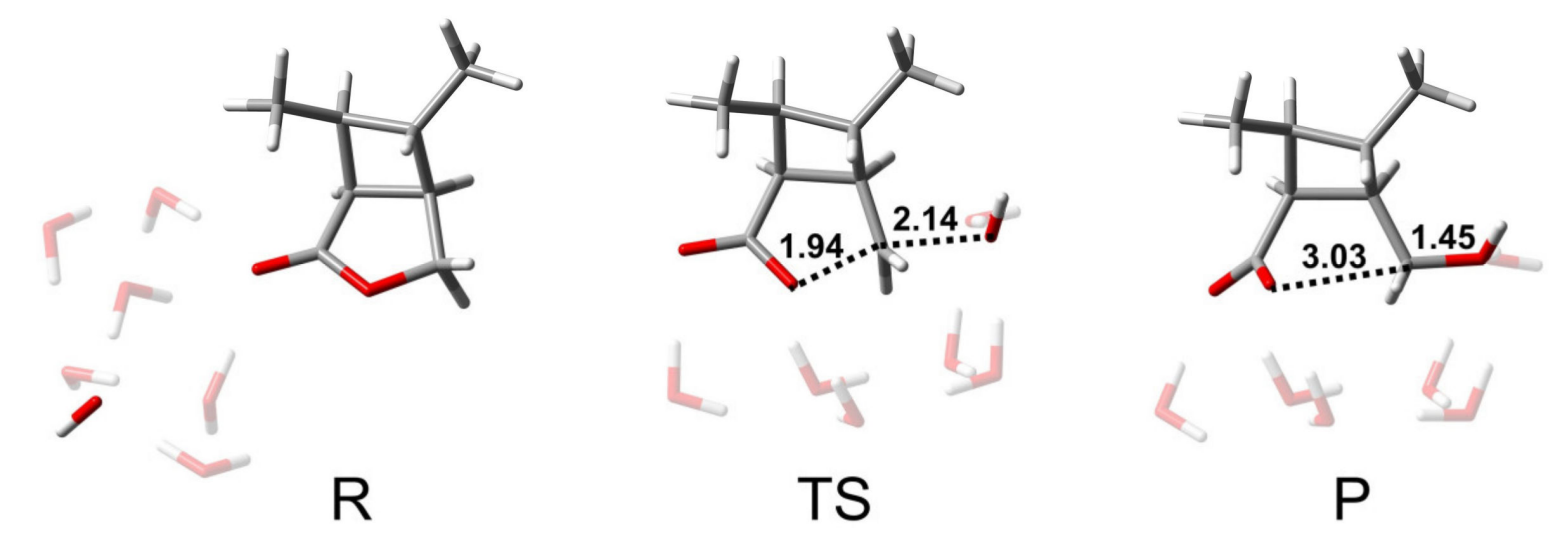
Structures of R, TS, and P of B_AL_2 reaction path of CBL with 6 H_2_O molecules. The unit of distance is Å.

**Figure 4:**
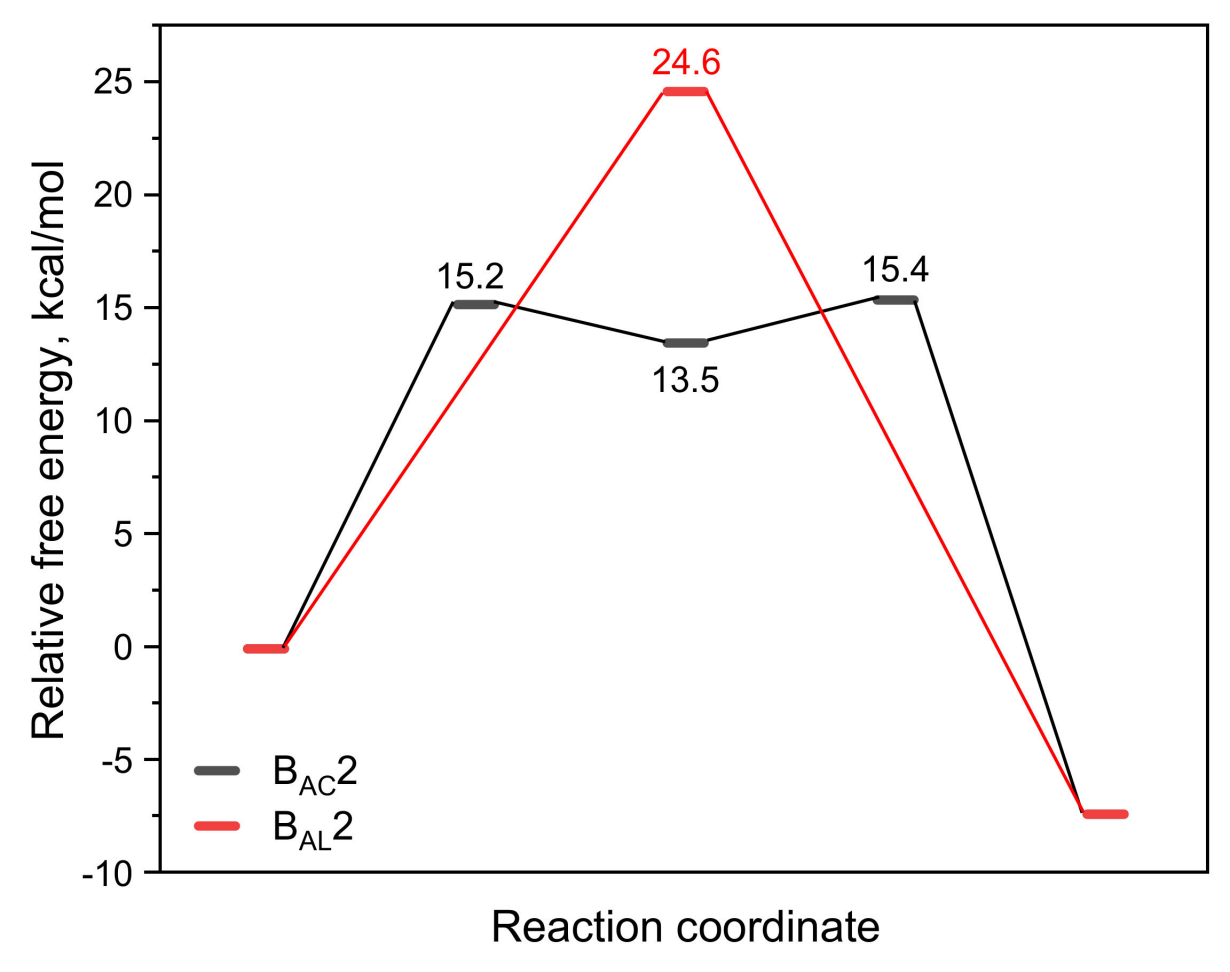
Variation in the relative free energy (Δ*G*) along the B_AC_2 and B_AL_2 reaction paths for the alkaline hydrolysis of CBL.

From the analysis of the B_AC_2 reaction pathway, it could be seen that the number of water molecules did not have a great influence on the hydrolysis mechanism and the energy barrier. Here, we used a model containing six water molecules (CBL + 6H_2_O + OH^−^) for the following discussion. Figure 3 shows the structures of R, TS, and P of the B_AL_2 reaction path. TS corresponds to the single-step hydrolysis reaction in which nucleophilic attack and alkyl–oxygen cleavage occur simultaneously (similar to S_N_2 nucleophilic substitution reaction mechanism). The structure of the transition state showed that there were two water molecules forming hydrogen bonds with OH^−^ and two water molecules forming hydrogen bonds with alkoxyl oxygen and carbonyl oxygen. The remaining two water molecules were not connected to CBL, as they were only hydrogen-bonding to several water molecules. The bond distances of C-O_nuc_ and C-O_lg_ were 2.14 and 1.94 Å. The activation energy barrier of the reaction was 24.6 kcal/mol, which was 9 kcal/mol higher than that of the B_AC_2 pathway (Figure 4), indicating that the alkaline hydrolysis reaction of CBL tended to proceed along B_AC_2. In addition, the C adjacent to ether O was sp^3^ hybrid and less positive charged than the ketonic C, which indicated that B_AL_2 is less likely to occur. We could directly obtain the stable product (P in Figure 3) through the B_AL_2 hydrolysis pathway without proton transfer happening.

### 3.2. Acidic Hydrolysis

**Scheme 3:**
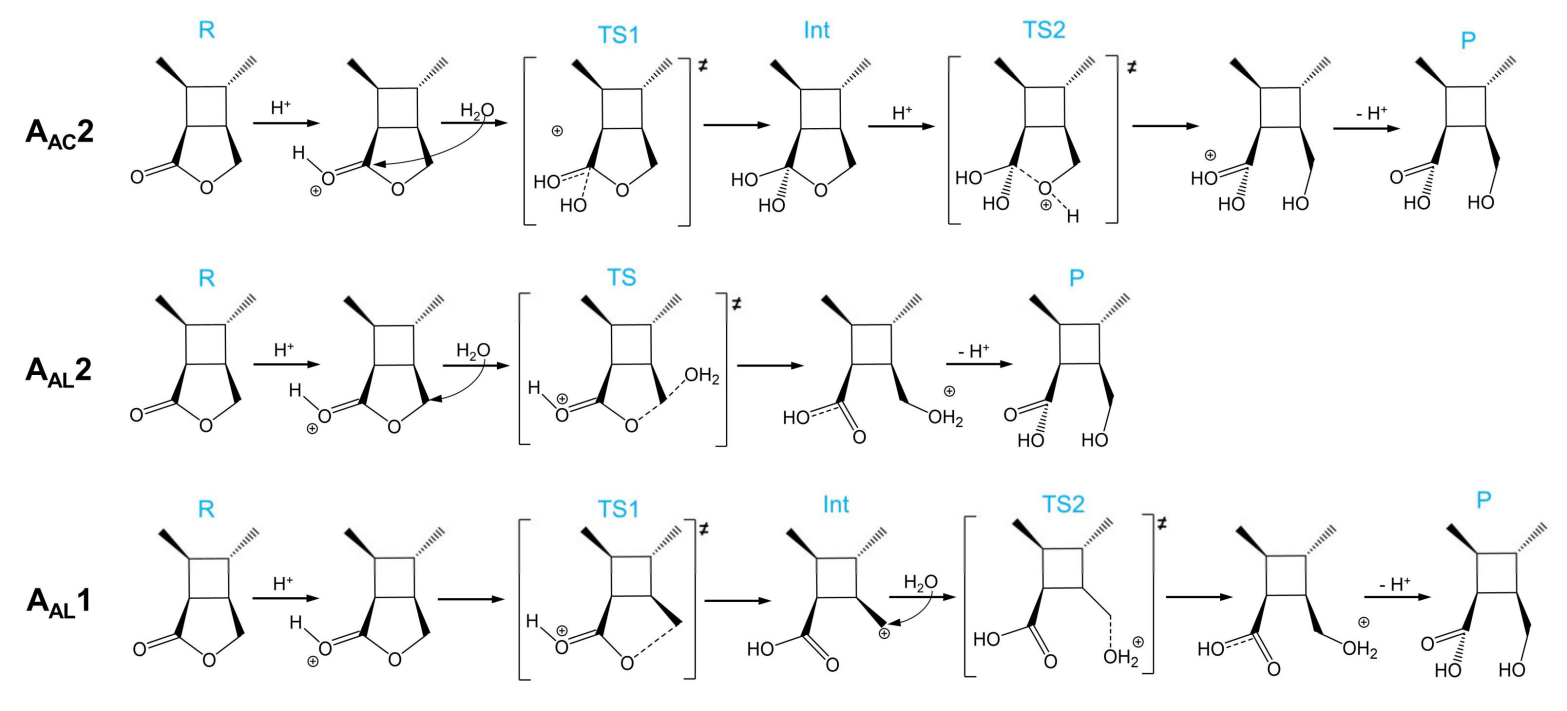
A_AC_2, A_AL_2, and A_AL_1 mechanisms of the acidic hydrolysis of CBL.

**Figure 5:**
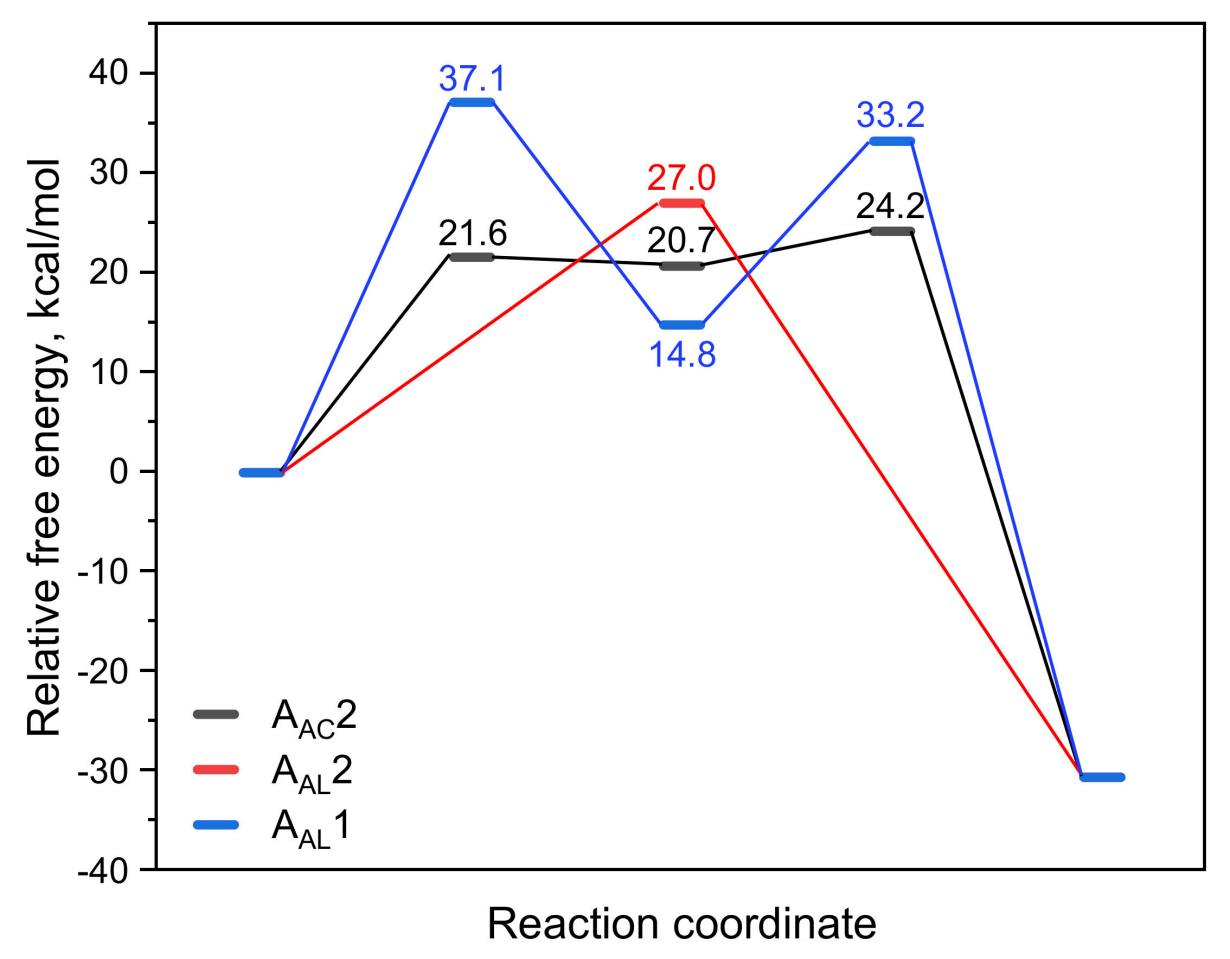
Variation in the relative free energy (Δ*G*) along the A_AC_2, A_AL_2, and A_AL_1 reaction paths for the acidic hydrolysis of CBL.

As shown in Scheme 3, there are three acid-catalyzed mechanisms of CBL hydrolysis: A_AC_2, A_AL_2, and A_AL_1. The A_AC_2 mechanism is generally considered to be the most favorable mechanism in acidic conditions: the carbonyl oxygen is protonated, one water molecule acts as a nucleophile to attack the acyl–carbon, and finally the acyl–oxygen bond is broken to form a product. The activation free energy of A_AC_2 was found to be 24.2 kcal/mol (Scheme 3 and Figure 5). Because the protonation and deprotonation processes in the reaction are not rate-limiting steps, we focused on the two transition states (TS1 and TS2) that affect the activation free energy. In the A_AL_2 mechanism, one water molecule attacks the alkyl–carbon, and the alkoxy bond is simultaneously broken to obtain the pre-product. The activation free energy of this mechanism was found to be 27.0 kcal/mol, which was slightly higher than that of A_AC_2. The least likely to occur was found to be the A_AL_1 mechanism. The energy barriers of both the cleavage step and the addition step were quite high, and the activation free energy of the entire reaction was 37.1 kcal/mol. The A_AL_1 and A_AL_2 mechanisms were obviously not feasible at room temperature.

We did not find the transition state of the A_AC_1 mechanism in our calculations, but we tried to fix the positions of the carbonyl carbon and the alkyl–oxygen to do a relaxed potential energy surface scan (Figure S4). The initial length of C···O(H) was 1.38 Å, and then scan steps of 0.02 Å were carried out (1.38–2.38 Å). It can be seen from the results that the rupture of the acyl–oxygen bond was accompanied by the protonation of the alkyl–oxygen. The distance of C-O changed from 1.38 to 2.38 Å, and the distance between alkyl–oxygen and H^+^ changed from 1.76 to 0.99 Å. The electronic energy barrier (Δ*E*) required for the A_AC_1 mechanism was about 28 kcal/mol without any thermal correction.

Here, we use CBL + 5H_2_O + H_3_O^+^ as the model to discuss the following three mechanisms: A_AC_2, A_AL_2, and A_AL_1. These three mechanisms are all based on the protonation of the carbonyl oxygen. The calculation level and methods can be found in the computational details section.

#### 3.2.1. The A_AC_2 Mechanism

**Figure 6:**
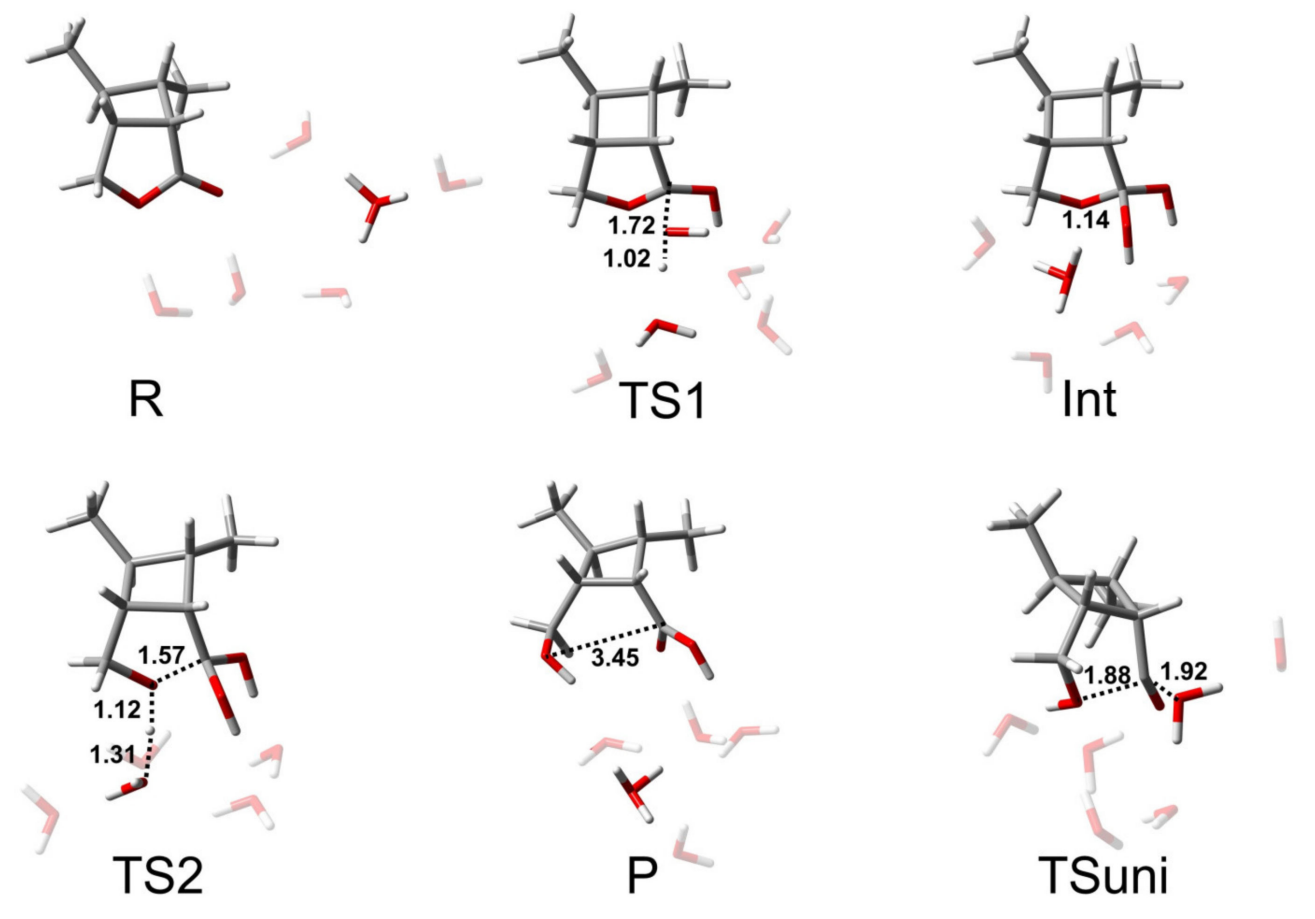
Structures of the stationary states of the A_AC_2 reaction path of CBL + 5H_2_O + H_3_O^+^. The unit of distance is Å.

The A_AC_2 mechanism is similar to B_AC_2, both of which are two-step reactions: the addition step and the elimination step. Figure 6 shows the key structures in the A_AC_2 reaction path. In the system, H^+^ ions are not independent; most of the H^+^ ions are present in H_3_O^+^ form. TS1 is the transition state of the addition reaction of one water attacking the carbon atom, and, at the same time, the proton of the nucleophile is transferred to an adjacent water molecule. The distance of C-O_nuc_ was found to be 1.72 Å, and the H-O_nuc_ bond in the nucleophile was elongated to 1.02 Å. The activation free energy of this step was found to be 21.6 kcal/mol (Figure 5). Int is the tetrahedral intermediate that lays at a high energy and easy to the next reaction. TS2 is the transition state when the acyl–oxygen bond is broken, and the cleavage of the acyl–oxygen bond simultaneously occurs with the protonation of the alkyl–oxygen. The C-O_lg_ bond length was found to be 1.57 Å, and the H-O_lg_ bond length was 1.12 Å. The activation free energy of the second step was 3.5 kcal/mol. Therefore, the elimination step was found to be the rate-limiting step. For this multiple reaction pathway, the calculated Gibbs energy of activation Δ*G^≠^* derived from experiment was 24.2 kcal/mol.

A single-step hydrolysis reaction (TS_uni_), in which nucleophilic addition and acyl–oxygen cleavage simultaneously take place, is shown in Figure 6. This reaction pathway was higher in activation free energy than two-step reaction by about 7 kcal/mol. The C-O_nuc_ bond length was 1.92 Å, and the C-O_lg_ bond length was 1.88 Å.

#### 3.2.2. The A_A__L_2 Mechanism

**Figure 7:**
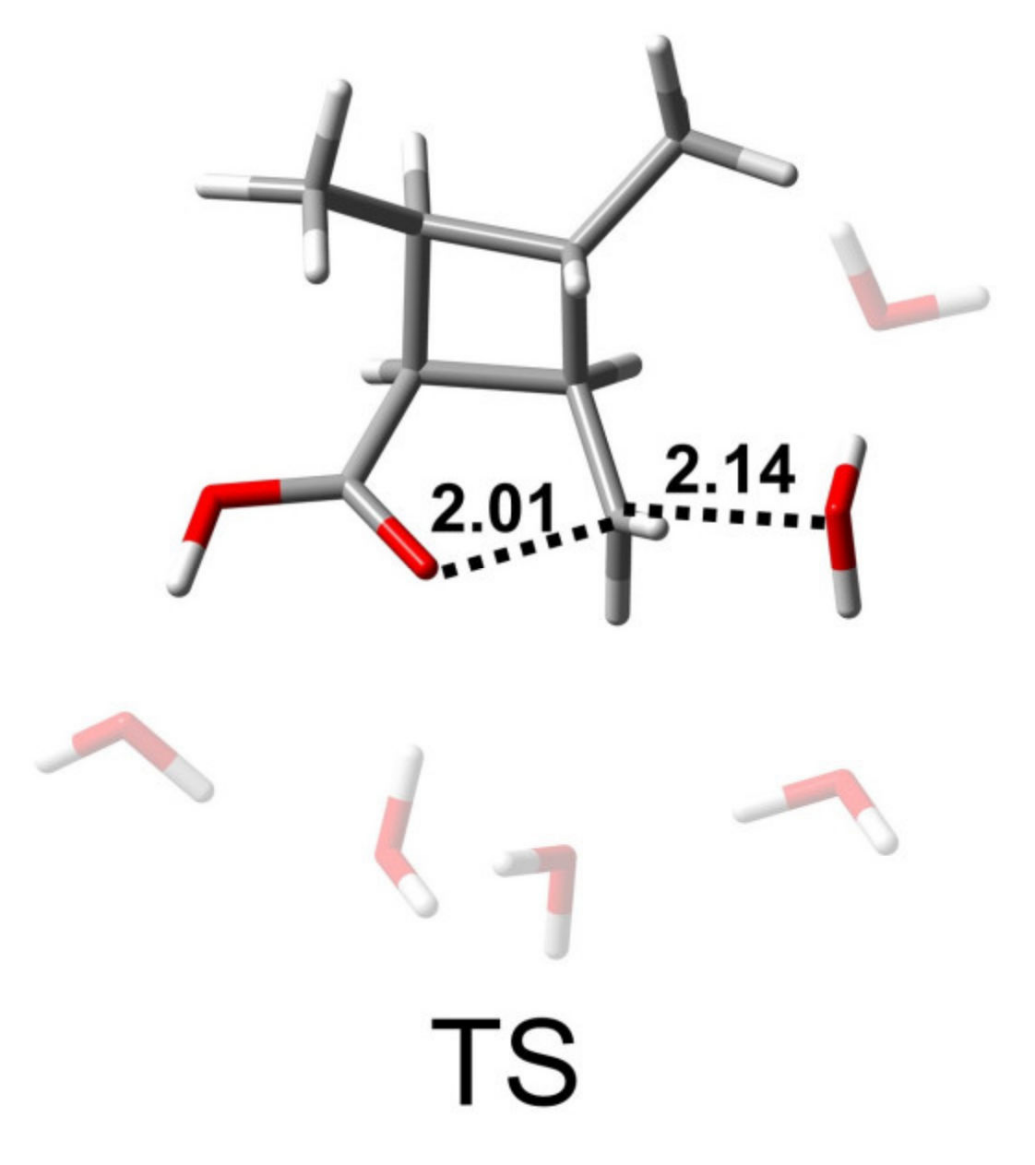
Structure of the transition state of the A_AL_2 reaction path of CBL + 5H_2_O + H_3_O^+^. The unit of distance is Å.

As shown in Scheme 3 and Figure 7, the A_AL_2 mechanism is a single-step hydrolysis reaction in which a water molecule attacks the alkyl–carbon and the alkoxy bond breaks. The bond lengths of C-O_nuc_ and C-O_lg_ were found to be 2.14 and 2.01 Å, respectively. Similarly, water molecules are distributed around the reaction site and form hydrogen bonds with key groups. The reaction energy barrier of this pathway was found to be 27.0 kcal/mol.

#### 3.2.3. The A_A__L_1 Mechanism

**Figure 8:**
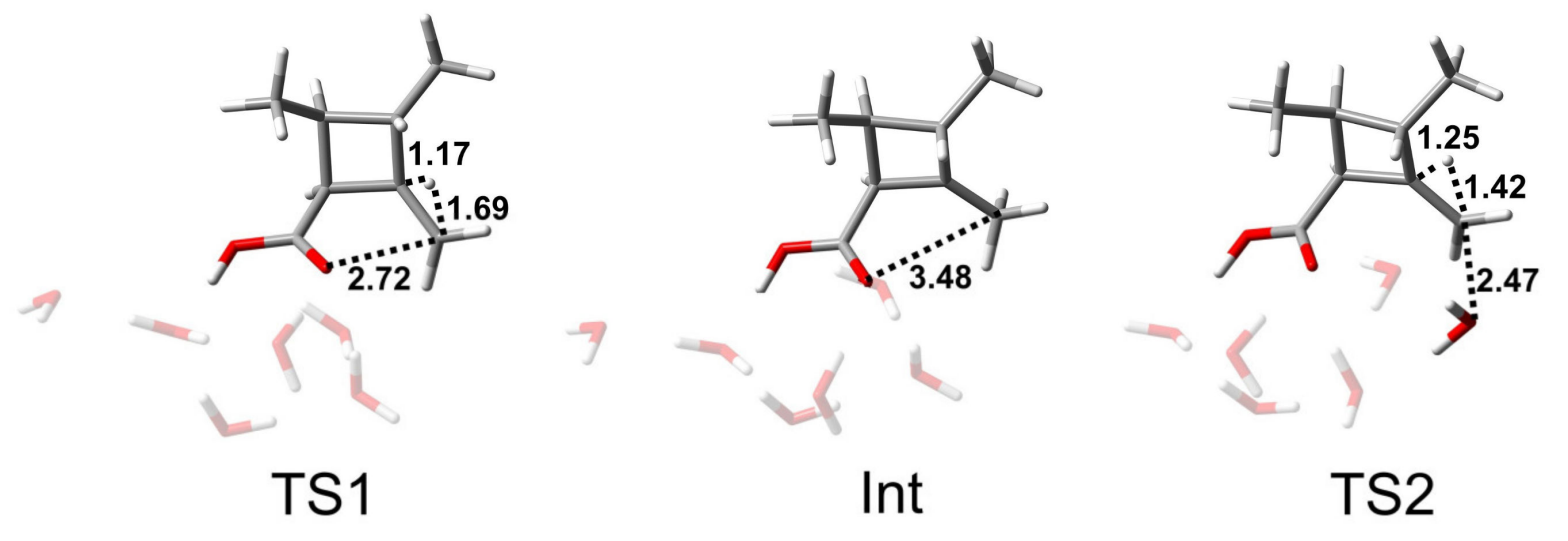
Structures of TS1, Int, and TS2 of the A_AL_1 reaction path of CBL + 5H_2_O + H_3_O^+^. The unit of distance is Å.

As shown in Scheme 3 and Figure 8, the A_AL_1 mechanism is a two-step hydrolysis reaction: the elimination step and the addition step. Firstly, the alkoxy bond is broken, and a proton on the cyclobutane transfers to the alkyl–carbon to obtain the intermediate (Int). The C-O_lg_ bond length of the intermediate was found to be 3.48 Å, indicating that the C-O_lg_ bond was completely broken and deformed, which facilitated the addition reaction of water molecules in the next step. The reaction energy barrier of the elimination step was 37.1 kcal/mol, which was higher than that of the addition step of 33.2 kcal/mol, so the first step was shown to be the rate-limiting step. TS2 is the transition state of the addition step. From the structure of TS2, it could be seen that the water molecule formed a bond with the alkyl–carbon, and the proton of the alkyl–carbon returned to the cyclobutane. The C-O_nuc_ bond length was found to be 2.47 Å, and the C-H_lg_ bond length was 1.42 Å.

## 4. Conclusions

Various mechanisms of a cyclobutane-fused lactone hydrolysis in alkaline and acidic conditions were studied using DFT-D3 and mixed implicit/explicit solvent models. This study indicates that it is highly possible for CBL to follow the B_AC_2 and A_AC_2 mechanisms in alkaline and acidic conditions, respectively. The B_AC_2 and A_AC_2 mechanisms are the two most important pathways in ester hydrolysis, and both of them are two-step reactions. We conducted a detailed discussion of the B_AC_2 mechanism, and the calculation results showed that adding an increasing number of explicit water molecules to the B_AC_2 reaction tightens the transition state geometries by introducing hydrogen-bonding interactions. When there was one water molecule or no water molecule in the model, activation free energies were remarkably lower than when more water molecules were added. Regardless of acidic or alkaline hydrolysis, the structures of the CBL hydrolysate remain unchanged, i.e., the initial “lactone group” remains cyclic after hydrolysis, which is beneficial to the reverse reaction (esterification reaction). These conclusions will help to guide future experiments and develop functional materials with degradability and stability.

## Figures and Tables

**Table 1:** Relative free energies and the corresponding activation free energies (Δ*G^≠^*) of the B_AC_2 reaction path of CBL with 0–6 H_2_O molecules. Free energies of TS1, Int, and TS2 are relative to the R of each pathway. Energies are presented in kcal/mol.

System	TS1	Int	TS2	Δ*G^≠^*
0 H_2_O	11.6	7.6	12.5	12.6
1 H_2_O	12.9	9.3	13.0	13.3
2 H_2_O	13.7	13.4	16.6	16.6
3 H_2_O	12.3	11.7	16.4	16.4
4 H_2_O	15.3	14.5	16.6	16.6
5 H_2_O	15.7	14.6	16.0	16.3
6 H_2_O	15.2	13.5	15.4	15.7

## Data Availability

The data presented in this study are available on request from the corresponding author.

## Supplementary Materials

The following are available online: Figure S1: Structures of the transition state of intramolecular proton transfer along the B_AC_2 reaction path without H_2_O or with one H_2_O molecule. Figure S2: The results of intrinsic reaction path calculation of CBL along B_AC_2 reaction path with 5 H_2_O molecules. Figure S3: The potential energy surface scan (PES) curves of deprotonation and protonation of CBL + 6H_2_O + OH^−^. Figure S4: The potential energy surface scan (PES) curves of acyl–oxygen cleavage of CBL + 5H_2_O + H_3_O^+^. Table S1: Free energies of TS1, Int1, and TS2 relative to R along the B_AC_2 reaction path of γ-butyrolactone. Table S2: Free energies of TS1, Int1, and TS2 relative to R along the A_AC_2 reaction path of γ-butyrolactone. Table S3: Comparison of C-O distances to the nucleophile (C-O_nuc_) and leaving group (C-O_lg_) at TS1, Int, TS2, and P of the B_AC_2 reaction path of CBL, with 0–6 H_2_O molecules. Table S4: Comparison of dihedral angles (C-C-C-C) to the cyclobutane at R, TS1, Int, TS2, and P of the B_AC_2 reaction path of CBL, with 0–6 H_2_O molecules. Cartesian coordinates and SCF energies of all optimized stationary points are provided.
