# Supplementary material for: Mechanisms of a Cyclobutane-Fused Lactone Hydrolysis in Alkaline and Acidic Conditions
Source: Molecules. 2021 Jun 9;26(12):3519. doi: 10.3390/molecules26123519 (PMC8226438; doi:10.3390/molecules26123519)
Supplement: Supplementary file 1 [file molecules-26-03519-s001.zip › molecules-1236527-supplementary.pdf]

Supplementary Materials

Mechanisms of a Cyclobutane-Fused Lactone Hydrolysis in Alkaline and Acidic Conditions

Zhangxia Wang and Haibo Ma

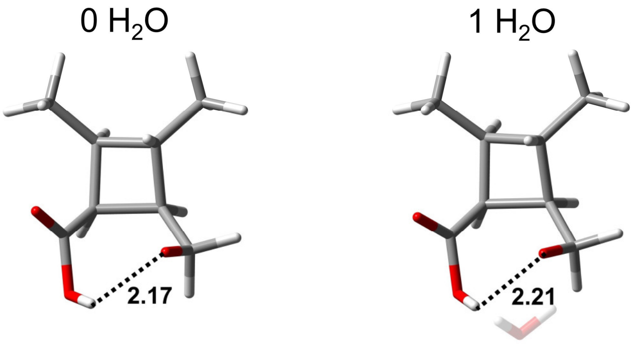

Figure S1. Structures of the transition state of intramolecular proton transfer along the $B_{AC}2$ reaction path, without H_2O or with one H_2O molecule. The unit of distance is Å.

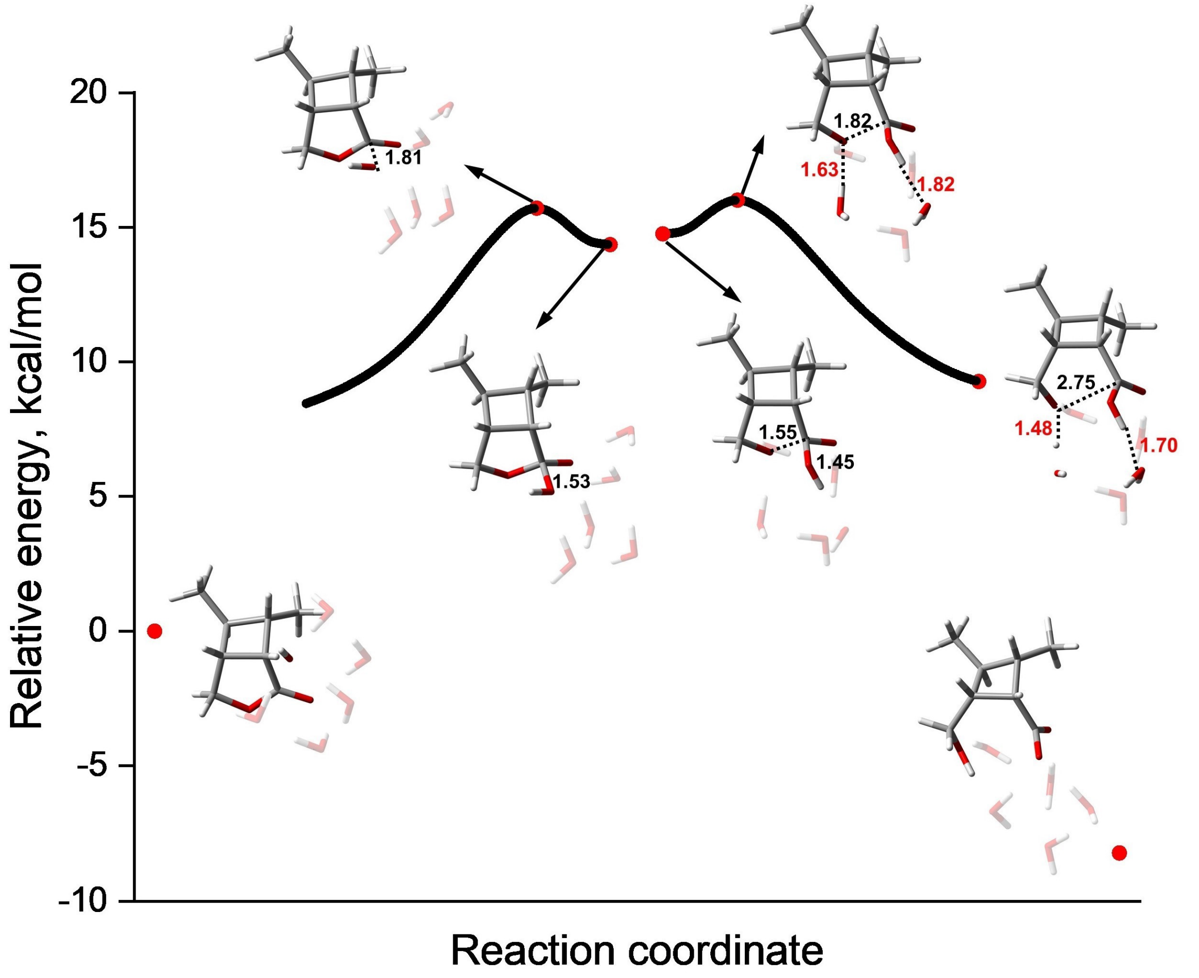

Figure S2. The results of intrinsic reaction path calculation of CBL along the $B_{AC}2$ reaction path with 5 H_2O molecules. The unit of distance is Å.

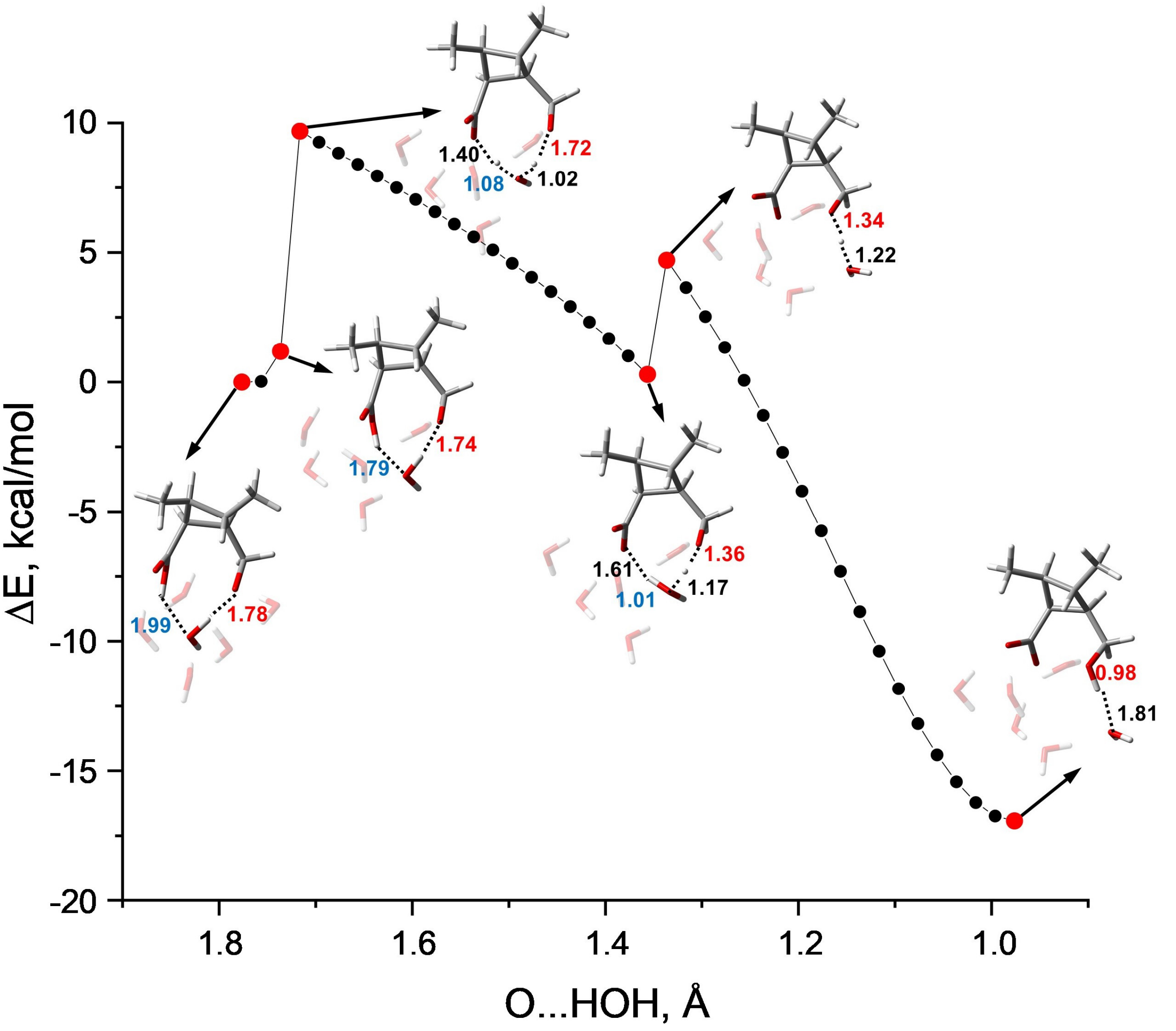

Figure S3. The potential energy surface scan (PES) curves of the deprotonation and protonation of CBL + 6H_2O + OH^-. O…HOH denotes the distance between the alkyl–oxygen and the hydrogen atom of H_2O, which was set as 1.78 Å when the energy of the system was zero. The unit of distance is Å.

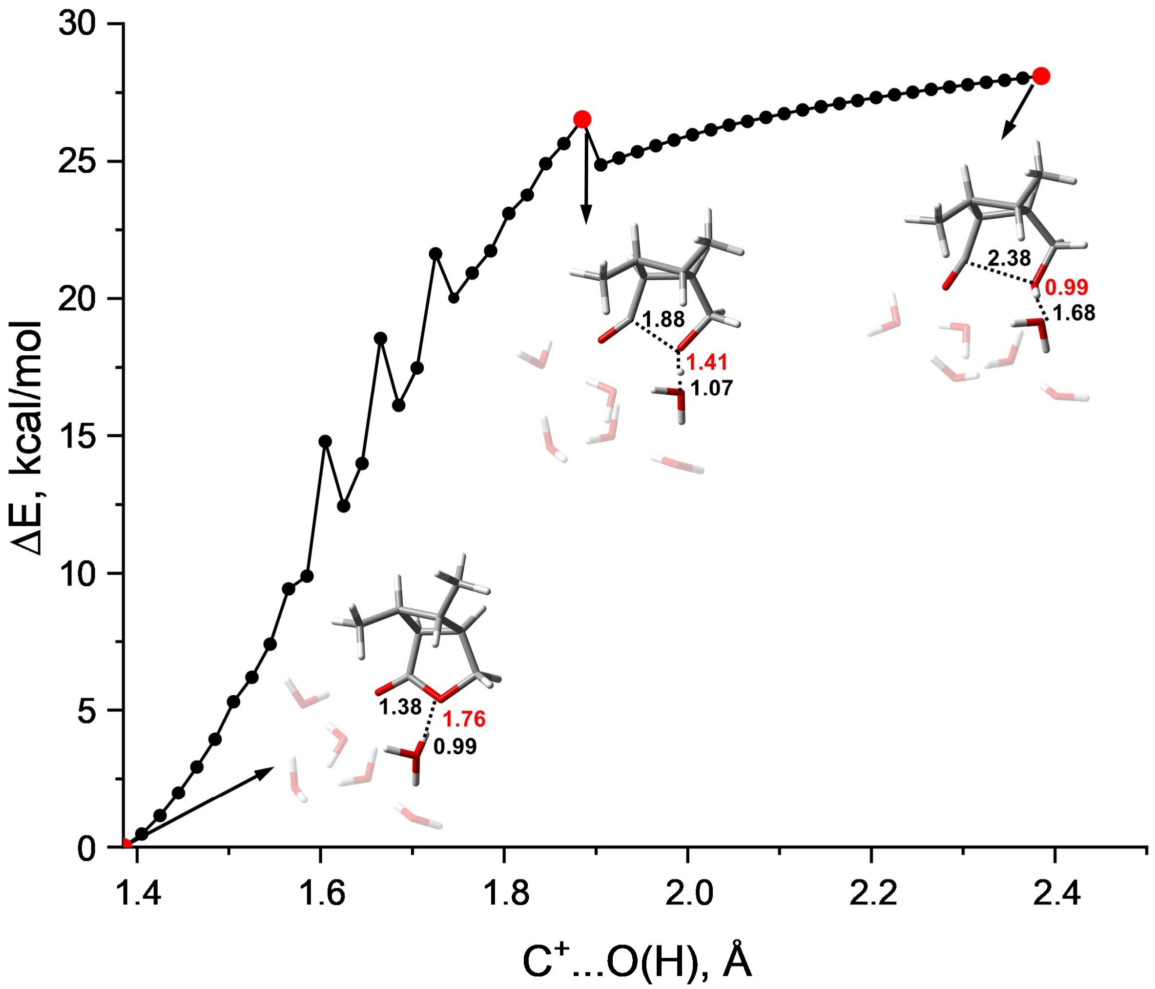

Figure S4. The potential energy surface scan (PES) curves of the acyl–oxygen cleavage of CBL + $5H_2O$ + H_3O^+. The C^+…O(H) distance was set as 1.38 Å when the energy of the system was zero. The unit of distance is Å.

Table S1. Free energies of TS1, Int1, and TS2 relative to R along the BAC2 reaction path of γ-butyrolactone. Microsolvation with 5 H_2O molecules was considered in SMD. The experimental value of activation free energy ($\Delta G^{\neq}_{exp}$) was 17.4–17.8 kcal/mol.[1] Energies are presented in kcal/mol.

Functional	Basis set	TS1	Int	TS2	$\Delta G^{\neq}_{calc}$
B3LYP	6-311+G(d)	18.5	12.9	17.7	18.7
	6-311++G(d,p)	20.3	13.1	18.2	20.3
	6-311++G(2df,pd)	20.9	13.6	19.3	21.0
B3LYP-D3	6-311+G(d)	13.9	8.7	11.7	14.0
	6-311++G(2df,pd)	13.9	8.0	11.8	13.9
M06-2X	6-311+G(d)	10.3	0.1	5.5	10.3
	6-311++G(2df,pd)	11.2	0.3	7.8	11.2

Table S2. Free energies of TS1, Int1, and TS2 relative to R along the $A_{AC}2$ reaction path of γ-butyrolactone. Microsolvation with 6 H_2O molecules was considered in SMD. The experimental value of activation free energy ($\Delta G^{\neq}_{exp}$) was 22.5–22.9 kcal/mol.[2] Energies are presented in kcal/mol.

Functional	Basis set	TS1	Int	TS2	$\Delta G^{\neq}_{calc}$
B3LYP	6-31++G(d,p)	22.6	17.8	22.9	23.2
	6-311++G(d,p)	23.5	19.4	23.8	24.1
B3LYP-D3	6-31++G(d,p)	22.4	17.8	20.6	22.5
	6-311++G(d,p)	22.6	18.3	20.5	22.7
M06-2X	6-311++G(d,p)	19.5	13.3	17.8	19.5

Table S3. Comparison of C-O distances to the nucleophile (C-O_{nuc}) and leaving group (C-O_{lg}) at TS1, Int, TS2, and P of the $B_{AC}2$ reaction path of CBL, with 0–6 H_2O molecules. All distances are given in Å.

**Distance**	**TS1**	**Int**		**TS2**	**P**
	**C-O_{nuc}**	**C-O_{nuc}**	**C-O_{lg}**	**C-O_{lg}**	**C-O_{lg}**
0 H_2O	1.99	1.49	1.50	2.07	3.37
1 H_2O	1.88	1.48	1.48	1.92	3.38
2 H_2O	1.79	1.54	1.47	1.85	3.04
3 H_2O	1.85	1.54	1.45	1.84	2.99
4 H_2O	1.88	1.45	1.52	1.88	3.10
5 H_2O	1.81	1.53	1.45	1.82	3.12
6 H_2O	1.81	1.47	1.51	1.93	2.96

Table S4. Comparison of dihedral angles (C-C-C-C) to the cyclobutane at R, TS1, Int, TS2, and P of the BAC2 reaction path of CBL, with 0–6 H_2O molecules. All angles are given in °.

**Dihedral Angles**	**R**	**TS1**	**Int**	**TS2**	**P**
0 H_2O	12.2	6.5	7.0	3.4	17.8
1 H_2O	12.1	7.6	6.4	4.9	17.6
2 H_2O	11.5	7.5	6.8	3.8	19.4
3 H_2O	11.8	5.9	4.6	1.9	19.3
4 H_2O	11.8	5.8	3.4	3.0	19.0
5 H_2O	12.2	6.6	6.1	5.6	20.8
6 H_2O	11.9	6.9	5.5	7.9	20.1

CARTESIAN COORDINATES AND SCF ENERGIES

BAC2_0H2O_R Cartesian Coordinates

Atomic Number	Coordinates (Angstroms)		
	X	Y	Z
8	0.132974	-1.663717	1.108474
6	-0.236979	-0.361237	-0.824440
6	0.692647	0.428752	0.127182
1	0.096290	-0.636021	-1.825659
1	1.541720	0.926279	-0.339480
6	-0.492178	1.378585	0.471675
1	-0.952492	1.074759	1.419553
6	-0.225056	2.871916	0.473223
1	0.250863	3.183796	-0.462173
1	-1.155622	3.438647	0.582328
1	0.437700	3.156061	1.297266
6	1.114697	-0.573446	1.189538
1	1.074904	-0.188902	2.208295
1	2.093085	-1.014174	0.995067
6	-0.571550	-1.605564	-0.045289
8	-1.360883	-2.484278	-0.334881
6	-2.765638	0.453513	-0.512440
1	-3.343371	1.373361	-0.376369
1	-3.185426	-0.080976	-1.370165
1	-2.915602	-0.166154	0.376694
6	-1.299565	0.785436	-0.720938
1	-1.180347	1.423850	-1.601334
8	4.319659	0.078092	-0.923388
1	5.206031	-0.199038	-0.656735

E(RB3LYP-D3) = -538.6332496 (Hartree)
Sum of electronic and thermal free energies = -538.477257 (Hartree)

BAC2_0H2O_TS1 Cartesian Coordinates

Atomic Number	Coordinates (Angstroms)		
	X	Y	Z
8	1.260815	-0.465422	-1.167041
6	0.151164	0.153663	0.835720
6	-0.478779	-1.154895	0.285962
1	0.446996	0.198142	1.880416
1	-0.712848	-1.933201	1.013801
6	-1.711777	-0.350661	-0.219035
1	-1.680167	-0.236958	-1.309248
6	-3.082223	-0.845711	0.210551
1	-3.124500	-0.984829	1.296191
1	-3.866289	-0.132269	-0.065355
1	-3.324668	-1.805800	-0.257972
6	0.488860	-1.632765	-0.791765
1	-0.008673	-1.994127	-1.693688
1	1.174394	-2.398831	-0.422921
6	1.350798	0.405520	-0.067529
8	1.914342	1.496060	-0.259726
6	-1.128478	2.210209	-0.330545
1	-2.148903	2.534510	-0.562788
1	-0.634986	3.023694	0.209862
1	-0.600988	2.070338	-1.277917
6	-1.165822	0.926899	0.483492
1	-1.715821	1.104525	1.413821
8	2.615047	-0.654837	1.043716
1	3.332359	-0.725160	0.399103

E(RB3LYP-D3) = -538.6225303 (Hartree)
Sum of electronic and thermal free energies = -538.458763 (Hartree)

BAC2_0H2O_Int Cartesian Coordinates

Atomic Number	Coordinates (Angstroms)		
	X	Y	Z
8	1.228395	-0.629498	-1.114296
6	0.160591	0.148326	0.873989
6	-0.532071	-1.151676	0.362494
1	0.389242	0.209185	1.938075
1	-0.844981	-1.876967	1.116889
6	-1.691401	-0.300690	-0.228394
1	-1.601007	-0.235067	-1.319560
6	-3.109870	-0.693959	0.148191
1	-3.215001	-0.785705	1.234747
1	-3.833887	0.053607	-0.194563
1	-3.390203	-1.655785	-0.295557
6	0.435921	-1.737206	-0.661944
1	-0.067565	-2.171306	-1.530287
1	1.081572	-2.501690	-0.213143
6	1.478906	0.257551	0.065059
8	1.959021	1.405992	-0.319604
6	-1.013087	2.227134	-0.404842
1	-2.023798	2.569713	-0.655917
1	-0.505551	3.044948	0.115659
1	-0.479531	2.046843	-1.340503
6	-1.098706	0.969203	0.445772
1	-1.671577	1.197598	1.352186
8	2.448412	-0.499486	0.902986
1	3.293970	-0.423532	0.437344

E(RB3LYP-D3) = -538.6308996 (Hartree)
Sum of electronic and thermal free energies = -538.465145 (Hartree)

BAC2_0H2O_TS2 Cartesian Coordinates

Atomic Number	Coordinates (Angstroms)		
	X	Y	Z
8	-1.072615	-0.869018	1.338549
6	-0.174590	0.180993	-0.871807
6	0.556413	-1.111229	-0.388533
1	-0.315455	0.235399	-1.954476
1	0.878709	-1.782486	-1.190987
6	1.708618	-0.238512	0.184587
1	1.685968	-0.241561	1.280834
6	3.115625	-0.541903	-0.304056
1	3.155826	-0.556038	-1.398856
1	3.830269	0.211874	0.045296
1	3.461290	-1.518246	0.053565
6	-0.307251	-1.824837	0.655841
1	0.349841	-2.391705	1.340305
1	-0.947741	-2.572716	0.150748
6	-1.552210	0.346113	-0.260894
8	-2.022371	1.380684	0.238117
6	0.854677	2.194978	0.604087
1	1.840937	2.563435	0.909129
1	0.306876	3.034243	0.166308
1	0.322097	1.881516	1.504326
6	1.030084	1.044437	-0.375916
1	1.578700	1.409182	-1.251295
8	-2.431920	-0.538393	-0.896908
1	-3.320268	-0.359318	-0.552807

E(RB3LYP-D3) = -538.6220567 (Hartree)

Sum of electronic and thermal free energies = -538.457324 (Hartree)

BAC2_1H2O_R Cartesian Coordinates

Atomic Number	Coordinates (Angstroms)		
	X	Y	Z
8	0.762524	-0.642345	1.354526
6	-0.258768	-0.194097	-0.724932
6	-0.414083	1.054156	0.176983
1	0.276931	-0.130918	-1.672310
1	-0.229157	2.015521	-0.302898
6	-1.923089	0.716209	0.358497
1	-2.077948	0.198442	1.312586
6	-2.924272	1.845589	0.205357
1	-2.768218	2.379582	-0.737736
1	-3.951102	1.465487	0.210016
1	-2.832952	2.571005	1.020525
6	0.505721	0.803616	1.361851
1	0.062159	1.039780	2.328643
1	1.474409	1.294598	1.265070
6	0.419892	-1.197255	0.169099
8	0.657543	-2.367374	-0.059180
6	-2.481413	-1.676807	-0.619586
1	-3.570261	-1.563910	-0.615009
1	-2.219913	-2.363777	-1.430280
1	-2.192867	-2.145963	0.325722
6	-1.817903	-0.323637	-0.796413
1	-2.142364	0.134078	-1.735679
8	3.859061	-0.156986	0.243015
1	3.136531	-0.574520	0.728366
8	2.875437	1.536988	-1.468354
1	3.215750	0.861252	-0.778383
1	1.945969	1.690434	-1.263821

E(RB3LYP-D3) = -615.1125038 (Hartree)

Sum of electronic and thermal free energies = -614.933090 (Hartree)

BAC2_1H2O_TS1 Cartesian Coordinates

Atomic Number	Coordinates (Angstroms)		
	X	Y	Z
8	-0.696645	0.728561	1.461530
6	0.045795	0.158688	-0.721979
6	0.227106	-1.060565	0.224207
1	-0.452552	0.012245	-1.677308
1	-0.021445	-2.042008	-0.182706
6	1.753399	-0.769766	0.305159
1	2.006152	-0.307908	1.267131
6	2.697388	-1.925337	0.021193
1	2.445599	-2.411023	-0.927724
1	3.735641	-1.582269	-0.044674
1	2.646913	-2.685541	0.808380
6	-0.580635	-0.714153	1.469822
1	-0.084804	-0.994065	2.401321
1	-1.582511	-1.149737	1.447982
6	-0.715901	1.181894	0.120712
8	-0.687330	2.420813	-0.040719
6	2.279621	1.656154	-0.558926
1	3.367668	1.526786	-0.553605
1	2.034983	2.384816	-1.337847
1	1.990785	2.086724	0.403233
6	1.601877	0.316579	-0.798529
1	1.934053	-0.087880	-1.760999
8	-2.437585	0.615063	-0.367478
1	-2.947327	0.859697	0.416590
8	-2.923316	-1.918407	-0.974155
1	-2.709333	-0.971380	-0.717646
1	-2.256713	-2.467655	-0.545515

E(RB3LYP-D3) = -615.0978125 (Hartree)

Sum of electronic and thermal free energies = -614.912593 (Hartree)

BAC2_1H2O_Int Cartesian Coordinates

Atomic Number	Coordinates (Angstroms)		
	X	Y	Z
8	0.863014	-0.747914	-0.843587
6	-0.462268	-0.033825	1.001358
6	-1.214282	-1.139951	0.198514
1	-0.448100	-0.123763	2.087469
1	-1.763894	-1.885725	0.776697
6	-2.106656	-0.052665	-0.463673
1	-1.806718	0.108807	-1.506229
6	-3.611861	-0.243963	-0.383922
1	-3.928322	-0.429578	0.648284
1	-4.144571	0.644395	-0.740935
1	-3.940139	-1.095102	-0.990799
6	-0.152492	-1.755361	-0.709094
1	-0.524449	-1.993458	-1.709095
1	0.277249	-2.662688	-0.268889
6	0.989050	-0.066353	0.469255
8	1.690467	1.036535	0.345465
6	-1.034737	2.325219	-0.197064
1	-1.913209	2.857081	-0.580868
1	-0.523020	2.991839	0.503413
1	-0.360740	2.144267	-1.036995
6	-1.475776	1.026886	0.461604
1	-2.174718	1.264258	1.271898
8	1.657639	-1.048187	1.350915
1	2.576091	-1.086261	1.046427
8	4.046380	0.542677	-0.830510
1	3.865080	-0.080546	-1.544189
1	3.163590	0.721668	-0.396324

E(RB3LYP-D3) = -615.1034418 (Hartree)
Sum of electronic and thermal free energies = -614.918328 (Hartree)

BAC2_1H2O_TS2 Cartesian Coordinates

Atomic Number	Coordinates (Angstroms)		
	X	Y	Z
8	1.076365	-0.562828	-0.805812
6	-0.475650	0.455765	0.929425
6	-0.748149	-1.050436	0.624293
1	-0.540614	0.730196	1.984684
1	-1.042907	-1.655401	1.486444
6	-1.939474	-0.646310	-0.289258
1	-1.681093	-0.794365	-1.344552
6	-3.288555	-1.280949	0.005153
1	-3.557755	-1.157477	1.059897
1	-4.082822	-0.825328	-0.596712
1	-3.281398	-2.354786	-0.212588
6	0.467313	-1.622842	-0.104769
1	0.157351	-2.423156	-0.794046
1	1.168676	-2.071417	0.618503
6	0.910882	0.875170	0.454825
8	1.189067	1.923589	-0.166883
6	-1.724998	1.857998	-1.017571
1	-2.704776	1.880249	-1.508494
1	-1.507418	2.869978	-0.663988
1	-0.979481	1.600315	-1.772650
6	-1.753050	0.843702	0.116094
1	-2.533480	1.135048	0.827877
8	1.841719	0.430902	1.416666
1	2.708996	0.768263	1.146909
8	3.701161	-0.878500	-0.904769
1	2.705288	-0.756459	-0.860556
1	4.015011	-0.843554	0.006506

E(RB3LYP-D3) = -615.097394 (Hartree)
Sum of electronic and thermal free energies = -614.912390 (Hartree)

BAC2_2H2O_R Cartesian Coordinates

Atomic Number	Coordinates (Angstroms)		
	X	Y	Z
8	-0.658519	-0.871926	0.582909
6	1.577592	-1.251334	-0.052028
6	1.571588	-0.411150	1.248630
1	2.070806	-2.222971	-0.084854
1	2.294135	-0.704185	2.009603
6	1.986581	0.843710	0.425666
1	1.108276	1.465870	0.219367
6	3.118150	1.697751	0.965508
1	3.996446	1.084405	1.192063
1	3.418458	2.458818	0.237924
1	2.822470	2.213188	1.885237
6	0.137544	-0.466736	1.750430
1	-0.260114	0.490995	2.083236
1	-0.022676	-1.225544	2.517018
6	0.119736	-1.410320	-0.386770
8	-0.380823	-1.914692	-1.370678
6	1.801779	0.369269	-2.167055
1	2.353371	1.256352	-2.494256
1	1.948034	-0.412207	-2.918953
1	0.738945	0.628147	-2.151667
6	2.289449	-0.070176	-0.798732
1	3.358201	-0.300374	-0.835581
8	-4.243461	1.385383	-0.283015
1	-4.613946	1.250285	-1.163945
8	-1.679723	1.917328	-0.522367
1	-2.668652	1.713987	-0.445588
1	-1.221081	1.098342	-0.296053
8	-3.593793	-0.956871	0.754961
1	-3.858105	-0.071543	0.350056
1	-2.628530	-1.003423	0.688013

E(RB3LYP-D3) = -691.5886053 (Hartree)

Sum of electronic and thermal free energies = -691.388735 (Hartree)

BAC2_2H2O_TS1 Cartesian Coordinates

Atomic Number	Coordinates (Angstroms)		
	X	Y	Z
8	0.155379	0.113405	1.835976
6	-0.153528	-0.191947	-0.501635
6	-0.911752	1.082693	-0.035702
1	0.495835	-0.123728	-1.371442
1	-0.867810	1.951566	-0.694499
6	-2.256266	0.301680	-0.094151
1	-2.594436	0.042191	0.916301
6	-3.394946	0.935336	-0.874648
1	-3.068013	1.220013	-1.880570
1	-4.236785	0.242500	-0.981151
1	-3.766947	1.837847	-0.377384
6	-0.373967	1.372114	1.361402
1	-1.145242	1.687828	2.066902
1	0.427914	2.114724	1.349013
6	0.656106	-0.624491	0.726007
8	1.005564	-1.804284	0.979785
6	-1.817283	-2.288233	-0.196217
1	-2.860849	-2.555181	-0.397524
1	-1.183988	-3.055592	-0.651752
1	-1.665163	-2.329075	0.884995
6	-1.523487	-0.903679	-0.751356
1	-1.711331	-0.902844	-1.830800
8	2.138567	0.316898	0.357710
1	2.600234	0.326790	1.207046
8	2.074238	2.687278	-0.897725
1	2.066718	1.821827	-0.407231
1	1.190388	3.058279	-0.791102
8	3.288561	-1.375996	-1.379989
1	2.887972	-0.743493	-0.724456
1	2.783760	-2.192884	-1.284602

E(RB3LYP-D3) = -691.5706696 (Hartree)
Sum of electronic and thermal free energies = -691.366966 (Hartree)

BAC2_2H2O_Int Cartesian Coordinates

Atomic Number	Coordinates (Angstroms)		
	X	Y	Z
8	0.092129	0.126916	1.866196
6	-0.130267	-0.184112	-0.486633
6	-0.888601	1.102705	-0.044824
1	0.517104	-0.114694	-1.360000
1	-0.817308	1.970718	-0.703820
6	-2.239099	0.337470	-0.138538
1	-2.616139	0.097778	0.863114
6	-3.342232	0.972919	-0.967694
1	-2.976820	1.239353	-1.965441
1	-4.188086	0.288257	-1.094711
1	-3.721456	1.886799	-0.497137
6	-0.390109	1.388306	1.368828
1	-1.178170	1.733707	2.042719
1	0.422605	2.122376	1.370814
6	0.710224	-0.598729	0.743902
8	0.948217	-1.833112	1.035572
6	-1.839997	-2.255311	-0.185646
1	-2.882639	-2.500823	-0.418801
1	-1.210892	-3.045024	-0.606985
1	-1.725710	-2.282150	0.899998
6	-1.500177	-0.885883	-0.753362
1	-1.663999	-0.902741	-1.836973
8	2.024744	0.170631	0.496327
1	2.545011	0.065251	1.305910
8	2.197031	2.578540	-0.828741
1	2.102841	1.735220	-0.332443
1	1.308065	2.949583	-0.881716
8	3.262671	-1.255632	-1.524556
1	2.848101	-0.727186	-0.806194
1	2.760706	-2.079366	-1.552905

E(RB3LYP-D3) = -691.5723884 (Hartree)
Sum of electronic and thermal free energies = -691.367324 (Hartree)

BAC2_2H2O_TS2 Cartesian Coordinates

Atomic Number	Coordinates (Angstroms)		
	X	Y	Z
8	-0.595374	-0.686954	0.736761
6	0.855893	0.556287	-0.875779
6	1.226592	-0.953724	-0.744700
1	0.891422	0.955519	-1.891684
1	1.527458	-1.452301	-1.670263
6	2.416736	-0.579418	0.183234
1	2.210437	-0.877732	1.218171
6	3.795077	-1.070002	-0.228708
1	4.013736	-0.797519	-1.267047
1	4.577785	-0.633506	0.401827
1	3.872409	-2.160023	-0.147910
6	0.060073	-1.658636	-0.051408
1	0.420958	-2.485115	0.577394
1	-0.629609	-2.087891	-0.795620
6	-0.557034	0.807223	-0.347615
8	-0.896172	1.786430	0.369543
6	2.024558	1.783539	1.231114
1	3.004149	1.808389	1.722604
1	1.734975	2.815934	1.015146
1	1.304193	1.377198	1.944220
6	2.113897	0.932404	-0.027062
1	2.862006	1.373410	-0.695364
8	-1.454319	0.414617	-1.360871
1	-2.369740	0.584667	-1.047570
8	-3.089627	-1.312568	1.275908
1	-2.126363	-1.095224	1.066824
1	-3.252696	-2.196545	0.925399
8	-4.106145	0.547941	-0.434100
1	-3.863012	-0.156576	0.213643
1	-4.659784	0.125536	-1.102145

E(RB3LYP-D3) = -691.570077 (Hartree)

Sum of electronic and thermal free energies = -691.362274 (Hartree)

BAC2_3H2O_R Cartesian Coordinates

Atomic Number	Coordinates (Angstroms)		
	X	Y	Z
8	0.210581	0.086505	0.967701
6	-1.240750	0.043864	-0.889407
6	-1.721096	1.149922	0.081470
1	-0.954241	0.287480	-1.912743
1	-2.017152	2.092596	-0.377683
6	-2.914708	0.241758	0.502157
1	-2.685350	-0.263831	1.447709
6	-4.293800	0.870654	0.564868
1	-4.526702	1.393995	-0.368280
1	-5.066073	0.111312	0.725740
1	-4.362877	1.595777	1.382479
6	-0.589029	1.314626	1.082807
1	-0.913705	1.380996	2.120468
1	0.078133	2.144145	0.846989
6	-0.071614	-0.573141	-0.171695
8	0.574717	-1.551413	-0.502672
6	-2.620134	-2.204768	-0.457267
1	-3.642024	-2.541198	-0.255232
1	-2.252512	-2.755707	-1.328317
1	-2.003286	-2.483218	0.402476
6	-2.602562	-0.706008	-0.694034
1	-3.241743	-0.451663	-1.544607
8	2.980803	1.572272	0.528245
1	2.327889	1.048483	1.009813
8	3.096405	-2.261328	0.679104
1	2.223284	-1.988966	0.346100
1	3.722420	-1.556219	0.402681
8	4.769195	-0.178480	-0.077704
1	4.084542	0.549416	0.170293
1	5.491213	-0.107992	0.558103
8	1.747662	1.947375	-1.758923
1	2.218140	1.797429	-0.872486
1	0.807292	2.006272	-1.552895

E(RB3LYP-D3) = -768.0598828 (Hartree)
Sum of electronic and thermal free energies = -767.837208 (Hartree)

BAC2_3H2O_TS1 Cartesian Coordinates

Atomic Number	Coordinates (Angstroms)		
	X	Y	Z
8	0.568027	-0.269440	1.001983
6	-0.868230	0.127562	-0.838493
6	-1.415060	0.875633	0.410106
1	-0.789114	0.666742	-1.779158
1	-1.804988	1.882143	0.252569
6	-2.507054	-0.218251	0.589053
1	-2.271044	-0.867127	1.440856
6	-3.947430	0.255270	0.682591
1	-4.194485	0.921942	-0.150608
1	-4.644582	-0.589105	0.652345
1	-4.127850	0.803932	1.613411
6	-0.270988	0.836178	1.418141
1	-0.595744	0.628454	2.439082
1	0.324704	1.751705	1.407920
6	0.500996	-0.384555	-0.396261
8	1.087815	-1.375355	-0.904744
6	-1.830943	-2.382563	-0.735386
1	-2.777005	-2.903582	-0.551085
1	-1.431489	-2.743886	-1.687738
1	-1.131364	-2.674286	0.052122
6	-2.064575	-0.880353	-0.748446
1	-2.778699	-0.632974	-1.541143
8	1.512471	1.103527	-0.825269
1	1.699422	0.932722	-1.757589
8	3.276660	-2.219442	0.482346
1	2.998454	-2.478998	1.368968
1	2.452522	-1.950643	0.004024
8	3.824640	0.584779	0.498181
1	3.793655	-0.386556	0.580481
1	2.986356	0.816174	0.020040
8	0.496962	3.580374	-0.724364
1	-0.262988	3.570145	-0.130607
1	0.841339	2.644127	-0.746788

E(RB3LYP-D3) = -768.0444923 (Hartree)
Sum of electronic and thermal free energies = -767.817613 (Hartree)

BAC2_3H2O_Int Cartesian Coordinates

Atomic Number	Coordinates (Angstroms)		
	X	Y	Z
8	0.574308	-0.217724	1.013025
6	-0.870623	0.139563	-0.831660
6	-1.443360	0.863167	0.424521
1	-0.856034	0.692810	-1.770306
1	-1.864976	1.859556	0.278542
6	-2.500263	-0.263896	0.595076
1	-2.262378	-0.896263	1.458975
6	-3.956694	0.165440	0.655072
1	-4.207144	0.817101	-0.189105
1	-4.627195	-0.700174	0.617765
1	-4.174144	0.716377	1.576752
6	-0.298893	0.848977	1.435647
1	-0.623858	0.626219	2.454498
1	0.253362	1.794341	1.440022
6	0.571648	-0.248684	-0.438347
8	1.124421	-1.323100	-0.923606
6	-1.727383	-2.416620	-0.677350
1	-2.656887	-2.961352	-0.475284
1	-1.319728	-2.792123	-1.620468
1	-1.016086	-2.663718	0.113942
6	-2.014040	-0.923623	-0.727868
1	-2.738035	-0.728852	-1.526965
8	1.420972	0.989563	-0.775931
1	1.688506	0.867687	-1.697361
8	3.304121	-2.130622	0.384941
1	3.024982	-2.469914	1.243787
1	2.468816	-1.840528	-0.085993
8	3.845940	0.625189	0.599017
1	3.822966	-0.352367	0.620585
1	3.004714	0.862518	0.156620
8	0.384248	3.554125	-0.816506
1	-0.420050	3.576331	-0.284279
1	0.708744	2.626970	-0.769795

E(RB3LYP-D3) = -768.0474147 (Hartree)
Sum of electronic and thermal free energies = -767.818640 (Hartree)

BAC2_3H2O_TS2 Cartesian Coordinates

Atomic Number	Coordinates (Angstroms)		
	X	Y	Z
8	0.264262	0.494392	0.813061
6	-1.222016	-0.650440	-0.823158
6	-1.388904	0.900472	-0.828550
1	-1.262728	-1.121996	-1.807373
1	-1.535609	1.364229	-1.807919
6	-2.692959	0.761762	0.007277
1	-2.552843	1.148180	1.024013
6	-3.957117	1.351211	-0.597280
1	-4.105937	0.992254	-1.621613
1	-4.843242	1.073462	-0.015620
1	-3.911075	2.445422	-0.632161
6	-0.195082	1.491443	-0.075887
1	-0.489204	2.394867	0.477212
1	0.601698	1.781955	-0.779836
6	0.113029	-1.044178	-0.191809
8	0.279860	-2.015780	0.593861
6	-2.624512	-1.494175	1.320744
1	-3.618456	-1.347156	1.759266
1	-2.454645	-2.571020	1.234866
1	-1.888187	-1.095045	2.021486
6	-2.559687	-0.789368	-0.026450
1	-3.321184	-1.221144	-0.685307
8	1.110546	-0.823316	-1.160042
1	1.973304	-1.138819	-0.812202
8	2.579386	1.224356	1.799325
1	1.689141	0.917721	1.435057
1	2.424702	2.084928	2.207235
8	3.946263	1.340945	-0.536684
1	3.468233	1.381110	0.328493
1	3.290272	1.565599	-1.208814
8	3.766566	-1.410880	-0.505097
1	3.971653	-0.452214	-0.583389
1	4.152514	-1.840424	-1.278116

E(RB3LYP-D3) = -768.039011 (Hartree)

Sum of electronic and thermal free energies = -767.811077 (Hartree)

BAC2_4H2O_R Cartesian Coordinates

Atomic Number	Coordinates (Angstroms)		
	X	Y	Z
8	0.250196	1.214107	-1.439804
6	0.781870	-0.253774	0.328934
6	1.550012	1.070439	0.548818
1	0.060753	-0.605725	1.064676
1	1.542545	1.459584	1.567116
6	2.873311	0.351208	0.151616
1	3.118601	0.570943	-0.894310
6	4.088166	0.569183	1.033465
1	3.848862	0.368736	2.082943
1	4.909545	-0.094397	0.743833
1	4.450826	1.600023	0.963089
6	0.954105	2.047230	-0.453949
1	1.692376	2.628517	-1.005251
1	0.208255	2.711253	-0.016742
6	0.089085	-0.051544	-0.989626
8	-0.541525	-0.861702	-1.641397
6	2.374781	-2.045982	-0.849545
1	3.402879	-2.419315	-0.804909
1	1.702358	-2.903196	-0.748711
1	2.222177	-1.612928	-1.842629
6	2.143445	-1.022269	0.246542
1	2.326119	-1.473072	1.226397
8	-2.473838	-1.055251	1.631917
1	-3.090178	-1.328817	2.321892
8	-1.311215	-3.008735	0.253318
1	-1.759080	-2.312901	0.818748
1	-0.942848	-2.510953	-0.492771
8	-1.894128	1.483119	1.759109
1	-0.942512	1.575166	1.887080
1	-2.094970	0.485955	1.756062
8	-3.812469	-0.420436	-0.616760
1	-3.463336	0.465723	-0.824735
1	-3.336524	-0.694037	0.216933
8	-2.579627	2.162234	-0.805564
1	-2.280964	1.981869	0.118151
1	-1.802681	2.013937	-1.360945

E(RB3LYP-D3) = -844.5401091 (Hartree)

Sum of electronic and thermal free energies = -844.292360 (Hartree)

BAC2_4H2O_TS1 Cartesian Coordinates

Atomic Number	Coordinates (Angstroms)		
	X	Y	Z
8	0.607022	-0.141120	0.714630
6	-1.012668	0.491484	-0.887923
6	-1.552850	0.798423	0.537538
1	-1.085773	1.258848	-1.654354
1	-2.090590	1.737983	0.671016
6	-2.461022	-0.462664	0.467669
1	-2.064423	-1.261419	1.105702
6	-3.940619	-0.262782	0.746598
1	-4.347986	0.547702	0.132780
1	-4.511497	-1.170384	0.522648
1	-4.117891	-0.007374	1.796939
6	-0.337504	0.678921	1.449268
1	-0.543312	0.164850	2.389190
1	0.130683	1.642934	1.656086
6	0.440194	0.088940	-0.665492
8	1.110406	-0.626884	-1.443872
6	-1.595960	-2.085452	-1.403094
1	-2.441772	-2.777451	-1.325771
1	-1.222905	-2.128016	-2.430577
1	-0.804442	-2.453993	-0.744850
6	-2.042758	-0.684549	-1.015012
1	-2.847567	-0.362784	-1.684190
8	1.237689	1.790206	-0.702723
1	1.390956	1.885935	-1.651668
8	1.139234	-2.743627	1.530622
1	0.853550	-1.847308	1.260929
1	0.694989	-3.350718	0.925724
8	3.279713	-1.765968	-0.197838
1	2.831942	-2.237159	0.524943
1	2.535557	-1.376831	-0.714535
8	3.604694	1.047693	0.433350
1	3.607812	0.077715	0.341554
1	2.749050	1.341233	0.027052
8	-0.128995	3.958123	0.050727
1	-0.853800	3.665572	0.615596
1	0.358440	3.129361	-0.220706

E(RB3LYP-D3) = -844.5169815 (Hartree)
Sum of electronic and thermal free energies = -844.267984 (Hartree)

BAC2_4H2O_Int Cartesian Coordinates

Atomic Number	Coordinates (Angstroms)		
	X	Y	Z
8	0.174066	-0.347871	-0.485550
6	-1.503503	0.323981	1.047698
6	-1.785357	-1.130103	0.554397
1	-1.635663	0.516995	2.112282
1	-2.128814	-1.851617	1.298662
6	-2.896397	-0.596380	-0.392793
1	-2.585236	-0.664763	-1.442282
6	-4.283165	-1.195788	-0.227090
1	-4.602715	-1.159104	0.820085
1	-5.024743	-0.649674	-0.820590
1	-4.307115	-2.243809	-0.545694
6	-0.496575	-1.568961	-0.139789
1	-0.672511	-2.130577	-1.060753
1	0.139162	-2.168727	0.521827
6	-0.037995	0.621191	0.661105
8	0.339660	1.824726	0.304482
6	-2.499002	1.941474	-0.876186
1	-3.416123	2.040713	-1.468444
1	-2.291316	2.913221	-0.419039
1	-1.680119	1.715272	-1.562637
6	-2.682985	0.842973	0.160760
1	-3.516504	1.116174	0.817477
8	0.733782	0.083168	1.769787
1	1.683133	0.230990	1.580908
8	2.361048	-0.560903	-2.035061
1	1.511771	-0.471820	-1.534700
1	2.141370	-0.866897	-2.923000
8	3.325500	-2.163033	-0.008153
1	3.044932	-1.695922	-0.824842
1	2.509114	-2.477415	0.401993
8	3.561322	0.312838	1.326784
1	3.593135	-0.570738	0.907388
1	3.446163	0.951210	0.590907
8	2.783924	1.960497	-0.790736
1	2.802431	1.230987	-1.434186
1	1.865097	1.929788	-0.396395

E(RB3LYP-D3) = -844.5229907 (Hartree)
Sum of electronic and thermal free energies = -844.269318 (Hartree)

BAC2_4H2O_TS2 Cartesian Coordinates

Atomic Number	Coordinates (Angstroms)		
	X	Y	Z
8	0.047527	0.728546	-0.767463
6	1.523804	-0.239169	1.023945
6	2.060052	1.100647	0.433283
1	1.640863	-0.345595	2.104464
1	2.522141	1.784493	1.150600
6	3.082529	0.311504	-0.431146
1	2.808970	0.351797	-1.492336
6	4.549331	0.672991	-0.263573
1	4.838596	0.656331	0.792971
1	5.196443	-0.032316	-0.796920
1	4.760624	1.676192	-0.650257
6	0.919980	1.756682	-0.343390
1	1.308490	2.314328	-1.207846
1	0.388606	2.478791	0.298182
6	0.039156	-0.419231	0.721619
8	-0.472605	-1.509327	0.345567
6	2.252636	-2.142139	-0.754202
1	3.151234	-2.441137	-1.306410
1	1.865717	-3.027909	-0.242824
1	1.503241	-1.823511	-1.481577
6	2.608735	-1.023038	0.213609
1	3.366112	-1.392315	0.913899
8	-0.696199	0.357784	1.623382
1	-1.644547	0.092901	1.578809
8	-2.220086	2.021102	-1.053883
1	-1.377119	1.473944	-0.951213
1	-2.253042	2.599439	-0.282096
8	-4.227810	0.264910	-0.806434
1	-3.552343	0.973682	-0.963404
1	-5.094964	0.622088	-1.030018
8	-3.397780	-0.266332	1.758067
1	-3.798608	-0.093882	0.877854
1	-3.786960	0.377611	2.362537
8	-2.642790	-2.008700	-1.275369
1	-3.288325	-1.285423	-1.181341
1	-1.894519	-1.772862	-0.684882

E(RB3LYP-D3) = -844.514776 (Hartree)
Sum of electronic and thermal free energies = -844.265939 (Hartree)

BAC2_5H2O_R Cartesian Coordinates

Atomic Number	Coordinates (Angstroms)		
	X	Y	Z
8	1.208092	-2.021389	1.061828
6	0.816429	-0.018883	-0.119065
6	1.909065	-0.819697	-0.867997
1	-0.124870	0.226375	-0.607873
1	1.803491	-0.866616	-1.951447
6	2.974955	0.202427	-0.373963
1	3.497662	-0.198112	0.502922
6	3.974075	0.729501	-1.386330
1	3.462347	1.116484	-2.273628
1	4.573018	1.542299	-0.962703
1	4.662049	-0.057446	-1.712629
6	1.911677	-2.194833	-0.217752
1	2.904190	-2.576559	0.019197
1	1.352418	-2.942810	-0.781831
6	0.545083	-0.851386	1.101218
8	-0.168541	-0.579975	2.054189
6	1.947371	1.846663	1.428485
1	2.769912	2.568819	1.410420
1	1.031175	2.386671	1.682792
1	2.151266	1.131776	2.231443
6	1.831886	1.155756	0.083169
1	1.637000	1.892543	-0.702306
8	-2.111694	0.554178	-2.089939
1	-2.406546	0.706906	-2.995518
8	-1.743329	1.830820	1.822976
1	-1.629728	2.138338	0.896727
1	-1.191670	1.034227	1.905575
8	-1.727431	-1.954718	-1.479579
1	-0.779898	-2.132149	-1.453521
1	-1.836980	-0.988582	-1.768804
8	-4.180315	0.345731	-0.369891
1	-3.830273	-0.326656	0.243254
1	-3.455849	0.465678	-1.043654
8	-2.804361	-1.742078	1.035704
1	-2.364095	-1.887198	0.164817
1	-2.144092	-1.298073	1.585430
8	-1.402595	2.701449	-0.792032
1	-0.447952	2.808063	-0.881092
1	-1.654442	1.886583	-1.340198

E(RB3LYP-D3) = -921.0132047 (Hartree)
Sum of electronic and thermal free energies = -920.745125 (Hartree)

BAC2_5H2O_TS1 Cartesian Coordinates

Atomic Number	Coordinates (Angstroms)		
	X	Y	Z
8	0.632401	-1.657271	0.478920
6	1.245430	0.401871	-0.526459
6	2.126400	-0.740356	-1.111496
1	0.762386	1.095268	-1.209659
1	2.315428	-0.717135	-2.185625
6	3.320271	-0.268028	-0.232380
1	3.474596	-0.954439	0.608571
6	4.639843	-0.009156	-0.938579
1	4.500134	0.659052	-1.795111
1	5.364112	0.459960	-0.263926
1	5.083559	-0.939335	-1.309844
6	1.445325	-2.028899	-0.661153
1	2.142317	-2.797517	-0.323948
1	0.798493	-2.453502	-1.435618
6	0.201742	-0.325981	0.322213
8	-0.373946	0.180175	1.325018
6	2.464812	1.289468	1.703833
1	3.466035	1.577598	2.043013
1	1.784774	2.115141	1.932101
1	2.149924	0.424182	2.292138
6	2.498435	0.974821	0.216984
1	2.836603	1.860915	-0.330731
8	-1.117657	-0.524532	-0.896341
1	-0.771510	-0.895969	-1.719226
8	-0.986395	2.845248	0.869196
1	-1.453028	2.692347	0.025089
1	-0.688830	1.946024	1.129064
8	-2.400213	-1.315371	2.476835
1	-2.892024	-1.556576	1.669057
1	-1.633040	-0.802131	2.138991
8	-3.392016	-1.664285	-0.128614
1	-3.512047	-2.531303	-0.533624
1	-2.507800	-1.312814	-0.438309
8	-4.736777	0.605883	-1.097955
1	-3.934644	1.138116	-1.269897
1	-4.396605	-0.243252	-0.752413
8	-2.226430	1.830160	-1.440836
1	-1.968356	2.116743	-2.324929
1	-1.765764	0.956135	-1.272735

E(RB3LYP-D3) = -920.9906693 (Hartree)

Sum of electronic and thermal free energies = -920.720048 (Hartree)

BAC2_5H2O_Int Cartesian Coordinates

Atomic Number	Coordinates (Angstroms)		
	X	Y	Z
8	0.651524	-1.600818	0.660344
6	1.260734	0.311229	-0.614508
6	2.170852	-0.893680	-1.006552
1	0.821288	0.901828	-1.417425
1	2.395205	-1.021119	-2.067121
6	3.332238	-0.288716	-0.168793
1	3.472598	-0.853142	0.760851
6	4.667865	-0.105670	-0.869264
1	4.542868	0.435139	-1.813625
1	5.365952	0.465860	-0.247885
1	5.136309	-1.069541	-1.096713
6	1.486136	-2.114353	-0.396927
1	2.183563	-2.827203	0.047801
1	0.868467	-2.647798	-1.130852
6	0.135292	-0.304611	0.248449
8	-0.373681	0.356758	1.245275
6	2.422019	1.501525	1.517056
1	3.417751	1.841248	1.824817
1	1.734293	2.344637	1.624926
1	2.105732	0.721444	2.212508
6	2.482998	0.989489	0.086279
1	2.833982	1.798570	-0.563969
8	-1.007637	-0.625972	-0.724213
1	-0.674483	-1.080682	-1.511581
8	-0.970971	2.908991	0.527011
1	-1.483882	2.706914	-0.275622
1	-0.687824	2.016180	0.847540
8	-2.489020	-0.681785	2.589185
1	-1.696291	-0.315824	2.119934
1	-2.978105	-1.152102	1.891679
8	-3.363114	-1.744336	0.109671
1	-3.435440	-2.675802	-0.130395
1	-2.474470	-1.436864	-0.187438
8	-4.762034	0.300797	-1.226462
1	-3.982885	0.854937	-1.429290
1	-4.395818	-0.474381	-0.757664
8	-2.306656	1.620629	-1.629980
1	-2.048194	1.794588	-2.543130
1	-1.796702	0.827725	-1.338432

E(RB3LYP-D3) = -920.9929097 (Hartree)
Sum of electronic and thermal free energies = -920.721854 (Hartree)

BAC2_5H2O_TS2 Cartesian Coordinates

Atomic Number	Coordinates (Angstroms)		
	X	Y	Z
8	-0.047552	-0.240225	0.893855
6	1.523866	-0.943814	-0.909796
6	2.070535	-1.214139	0.525835
1	1.689781	-1.743150	-1.634600
1	2.638805	-2.139488	0.650595
6	2.956304	0.059487	0.426888
1	2.544665	0.861498	1.050902
6	4.441131	-0.101593	0.706395
1	4.866987	-0.912611	0.105468
1	4.992595	0.814598	0.467567
1	4.626939	-0.335787	1.760528
6	0.894683	-1.112988	1.492048
1	1.209832	-0.714264	2.465189
1	0.443608	-2.101170	1.665021
6	0.017390	-0.684129	-0.867395
8	-0.551595	0.239828	-1.520788
6	2.087753	1.644220	-1.466920
1	2.941953	2.327470	-1.392469
1	1.723692	1.678770	-2.497672
1	1.296643	2.026406	-0.820239
6	2.527283	0.246276	-1.056396
1	3.339214	-0.075605	-1.718258
8	-0.642950	-1.924011	-0.837360
1	-1.607403	-1.758252	-0.948554
8	-2.417890	-0.669755	1.971734
1	-1.520063	-0.510009	1.551218
1	-2.664120	-1.570371	1.725766
8	-3.927741	0.734042	0.231949
1	-3.441310	0.298123	0.976059
1	-4.822516	0.919894	0.540631
8	-3.403119	-1.615658	-1.181490
1	-3.660191	-0.758868	-0.779886
1	-3.808385	-2.293878	-0.626622
8	-1.921465	2.444801	-0.682041
1	-2.727147	2.009498	-0.341292
1	-1.367935	1.695718	-1.000063
8	-0.206923	2.388115	1.558760
1	-0.831780	2.629081	0.847213
1	-0.023646	1.435372	1.372583

E(RB3LYP-D3) = -920.9922177 (Hartree)
Sum of electronic and thermal free energies = -920.719669 (Hartree)

BAC2_6H2O_R Cartesian Coordinates

Atomic Number	Coordinates (Angstroms)		
	X	Y	Z
8	1.535028	1.848652	-0.476692
6	2.079296	-0.020833	0.850648
6	3.323428	0.896144	0.770804
1	1.611050	-0.249446	1.808459
1	3.859146	1.055799	1.705889
6	4.000513	-0.132339	-0.183034
1	3.889611	0.193321	-1.224119
6	5.441830	-0.512885	0.097537
1	5.567126	-0.823747	1.139892
1	5.761357	-1.342699	-0.541240
1	6.117758	0.328988	-0.085160
6	2.820634	2.192226	0.153989
1	3.457698	2.591573	-0.634109
1	2.607223	2.967247	0.890706
6	1.085879	0.660812	-0.045250
8	-0.008081	0.244951	-0.395987
6	2.275034	-1.980231	-0.959154
1	3.017102	-2.668040	-1.376868
1	1.425731	-2.574452	-0.610709
1	1.925490	-1.336965	-1.772098
6	2.893774	-1.171723	0.166245
1	3.247523	-1.837918	0.958397
8	-4.389700	0.291061	0.945296
1	-5.315656	0.326871	1.212345
8	-1.002873	-2.177934	0.738722
1	-1.690911	-1.851345	1.364460
1	-0.577418	-1.382500	0.371506
8	-3.350748	2.650286	0.536637
1	-2.747276	2.846032	1.263205
1	-3.774212	1.751408	0.741820
8	-3.925580	-0.222591	-1.615797
1	-3.237613	0.444002	-1.810030
1	-4.165354	-0.062512	-0.654711
8	-1.970788	1.838766	-1.707024
1	-2.424587	2.217348	-0.919566
1	-1.220917	1.330189	-1.348972
8	-2.963656	-1.282006	2.434486
1	-2.578152	-0.743843	3.136082
1	-3.544869	-0.659197	1.876554
8	-2.623308	-2.658948	-1.498563
1	-2.037183	-2.564809	-0.719477
1	-3.097342	-1.800231	-1.571601

E(RB3LYP-D3) = -997.4824589 (Hartree)
Sum of electronic and thermal free energies = -997.194676 (Hartree)

BAC2_6H2O_TS1 Cartesian Coordinates

Atomic Number	Coordinates (Angstroms)		
	X	Y	Z
8	-1.063507	-1.151443	-1.372612
6	-1.531182	0.012674	0.642155
6	-2.585156	-1.114735	0.440160
1	-1.033409	0.102477	1.603945
1	-2.869891	-1.687626	1.323691
6	-3.631137	-0.053425	-0.008984
1	-3.776092	-0.091890	-1.095148
6	-4.977182	-0.066543	0.694364
1	-4.849469	-0.051670	1.782066
1	-5.577217	0.806733	0.416679
1	-5.553180	-0.962372	0.438155
6	-2.014582	-1.990063	-0.670957
1	-2.759494	-2.312429	-1.399928
1	-1.494494	-2.872709	-0.284949
6	-0.507540	-0.229355	-0.467650
8	0.202597	0.677500	-0.982927
6	-2.433600	2.202802	-0.636436
1	-3.356679	2.784209	-0.737866
1	-1.639070	2.888852	-0.328475
1	-2.175069	1.813634	-1.624151
6	-2.639663	1.083914	0.371457
1	-2.926807	1.516913	1.335690
8	0.673458	-1.281083	0.421041
1	0.213511	-2.028335	0.827096
8	1.315909	2.396589	0.851528
1	1.539546	1.729707	1.531874
1	0.820869	1.877809	0.176805
8	2.536717	0.176110	-2.337342
1	2.846654	-0.636951	-1.890947
1	1.645562	0.334637	-1.949731
8	2.978856	-2.007173	-0.676752
1	2.972284	-2.924165	-0.975219
1	2.073084	-1.810132	-0.299383
8	4.298246	-1.016417	1.597964
1	3.512440	-0.581061	1.983729
1	3.966972	-1.434345	0.779040
8	1.843579	0.117432	2.352244
1	1.419594	-0.053366	3.201527
1	1.352676	-0.422522	1.664638
8	3.632714	2.223295	-0.750277
1	3.340048	1.502871	-1.346946
1	2.879337	2.361643	-0.139765

E(RB3LYP-D3) = -997.4655154 (Hartree)

Sum of electronic and thermal free energies = -997.170471 (Hartree)

BAC2_6H2O_Int Cartesian Coordinates

Atomic Number	Coordinates (Angstroms)		
	X	Y	Z
8	0.235580	-0.818396	-0.454144
6	1.662083	1.082583	-0.588727
6	2.333150	-0.139155	-1.291091
1	1.691540	2.039176	-1.110559
1	2.858778	0.055579	-2.227614
6	3.258301	-0.342379	-0.059436
1	2.946341	-1.221827	0.516758
6	4.753819	-0.398965	-0.320074
1	5.079492	0.460979	-0.915339
1	5.319762	-0.388578	0.617807
1	5.030992	-1.306989	-0.866750
6	1.222409	-1.171116	-1.445414
1	1.546169	-2.193889	-1.242203
1	0.765349	-1.142557	-2.442059
6	0.186650	0.685055	-0.351019
8	-0.428121	1.061666	0.731827
6	2.280645	0.877271	2.039111
1	3.166062	0.687230	2.656956
1	1.820851	1.804383	2.392821
1	1.571541	0.065793	2.212115
6	2.697698	0.960575	0.578482
1	3.428186	1.769240	0.464782
8	-0.605223	1.118161	-1.517408
1	-0.139708	0.938718	-2.346290
8	-2.012828	-2.181782	-1.242523
1	-1.244885	-1.660446	-0.912218
1	-2.196315	-1.839288	-2.126468
8	-3.977412	-1.104902	0.275030
1	-3.342803	-1.636578	-0.262310
1	-4.804580	-1.598286	0.324462
8	-3.409756	1.092908	-1.406086
1	-3.710084	0.368939	-0.823828
1	-2.438768	0.992027	-1.480628
8	-2.077585	-0.457385	2.230708
1	-2.828758	-0.714884	1.663279
1	-1.490746	0.094034	1.652257
8	-0.034994	-2.338086	1.892046
1	-0.815820	-1.817835	2.172923
1	0.255520	-1.868488	1.086800
8	-2.458064	2.883635	0.617320
1	-3.016727	2.456776	-0.056546
1	-1.672704	2.287004	0.666709

E(RB3LYP-D3) = -997.4690015 (Hartree)

Sum of electronic and thermal free energies = -997.173154 (Hartree)

BAC2_6H2O_TS2 Cartesian Coordinates

Atomic Number	Coordinates (Angstroms)		
	X	Y	Z
8	0.266653	-1.155241	-0.173895
6	1.632867	0.860819	-0.948235
6	2.313226	-0.525777	-1.182520
1	1.691628	1.556598	-1.788716
1	2.889306	-0.600522	-2.108350
6	3.195066	-0.299926	0.076035
1	2.813818	-0.898428	0.911624
6	4.693184	-0.503778	-0.067987
1	5.088018	0.077479	-0.908403
1	5.226378	-0.188375	0.835581
1	4.937745	-1.556613	-0.247018
6	1.263140	-1.623930	-1.056202
1	1.716385	-2.549347	-0.673436
1	0.828339	-1.861888	-2.042425
6	0.151182	0.723224	-0.609231
8	-0.401527	1.305197	0.347776
6	2.276574	1.650483	1.565743
1	3.170748	1.705148	2.197321
1	1.827353	2.647344	1.538613
1	1.566934	0.976011	2.047542
6	2.673256	1.159034	0.181753
1	3.419188	1.844330	-0.235870
8	-0.664000	0.615816	-1.750019
1	-0.208923	0.149454	-2.465606
8	-1.942856	-2.424913	-0.804670
1	-1.111079	-1.917459	-0.547824
1	-2.169070	-2.121896	-1.692787
8	-3.821625	-1.184945	0.665701
1	-3.181499	-1.770811	0.188501
1	-4.580849	-1.722411	0.920147
8	-3.513210	0.605558	-1.481662
1	-3.760902	0.027914	-0.733885
1	-2.551869	0.486967	-1.595388
8	-2.056395	0.210819	2.295867
1	-2.744112	-0.295320	1.818064
1	-1.512425	0.624775	1.595450
8	0.130258	-1.587598	2.482581
1	-0.657205	-1.028752	2.627768
1	0.314905	-1.466422	1.517096
8	-2.650724	2.930018	-0.127489
1	-3.164990	2.256612	-0.612647
1	-1.811386	2.467035	0.065001

E(RB3LYP-D3) = -997.4637394 (Hartree)

Sum of electronic and thermal free energies = -997.170127 (Hartree)

BAL2_6H2O_TS Cartesian Coordinates

Atomic Number	Coordinates (Angstroms)		
	X	Y	Z
8	-0.395716	1.266140	-0.557900
6	-1.756351	0.111004	0.975063
6	-1.026134	-1.055200	0.255277
1	-1.681211	0.171715	2.061709
1	-0.745855	-1.852846	0.941236
6	-2.321959	-1.366833	-0.540356
1	-2.264959	-0.905795	-1.533195
6	-2.747773	-2.818318	-0.658847
1	-2.792200	-3.293361	0.326748
1	-3.737802	-2.905699	-1.118581
1	-2.042495	-3.388626	-1.273132
6	0.166780	-0.587335	-0.517424
1	0.183367	-0.600394	-1.592476
1	1.074511	-0.320489	-0.004743
6	-1.281825	1.410018	0.363270
8	-1.713632	2.522312	0.735399
6	-4.130913	0.519135	-0.182131
1	-4.964793	-0.039987	-0.619075
1	-4.542496	1.201219	0.567966
1	-3.684935	1.124175	-0.977557
6	-3.118611	-0.437702	0.421636
1	-3.590793	-1.034468	1.208096
8	1.122351	-2.488892	-0.738427
1	0.426484	-3.015723	-1.150569
8	1.725290	-2.664591	1.785153
1	2.168135	-3.502477	1.964456
1	1.434072	-2.691576	0.816634
8	3.181545	-1.020993	-1.640996
1	2.837647	-0.112285	-1.743114
1	2.401871	-1.604101	-1.443380
8	3.630702	-0.760000	1.115299
1	3.606012	-0.868529	0.139568
1	2.988809	-1.420892	1.457658
8	2.480418	1.717136	1.159224
1	1.530624	1.621987	1.307052
1	2.867373	0.811171	1.233421
8	2.319126	1.734721	-1.622053
1	2.457586	1.804067	-0.651566
1	1.355990	1.704488	-1.713708
8	0.331521	4.319468	-0.064888
1	-0.417398	3.743339	0.193164
1	0.980323	3.724070	-0.462033

E(RB3LYP-D3) = -997.4476247 (Hartree)

Sum of electronic and thermal free energies = -997.155440 (Hartree)

AAC2_6H2O_R Cartesian Coordinates

Atomic Number	Coordinates (Angstroms)		
	X	Y	Z
8	-1.594800	1.581003	-0.166197
6	-1.725016	-0.684986	-0.785761
6	-3.102267	-0.028810	-1.051060
1	-1.150972	-1.136651	-1.594405
1	-3.582759	-0.291713	-1.992006
6	-3.678818	-0.774161	0.189137
1	-3.698670	-0.100129	1.053128
6	-5.016783	-1.473428	0.034815
1	-5.008526	-2.135388	-0.837127
1	-5.245829	-2.078469	0.917854
1	-5.827373	-0.749548	-0.097108
6	-2.863517	1.466264	-0.913407
1	-3.617404	1.990139	-0.327829
1	-2.704775	1.970356	-1.866954
6	-0.927927	0.422092	-0.164096
8	0.194840	0.351280	0.318865
6	-1.751119	-1.922291	1.588994
1	-2.418822	-2.528925	2.208596
1	-0.806366	-2.463394	1.483860
1	-1.552453	-0.989734	2.125343
6	-2.398994	-1.660546	0.241291
1	-2.581813	-2.604275	-0.279414
1	4.178346	-0.145053	0.472938
8	4.130944	-1.172612	0.486432
1	4.687340	-1.533695	-0.276991
1	3.158979	-1.469758	0.353705
8	1.845590	2.429439	1.114393
1	1.742115	2.623702	2.054917
1	1.208356	1.709098	0.922021
8	0.078184	4.004473	-0.513751
1	0.789380	3.637309	0.041695
1	-0.592024	3.305008	-0.487788
8	1.712678	-1.939219	0.130410
1	1.106514	-1.171179	0.218893
1	1.435245	-2.567563	0.809784
8	4.289619	1.375751	0.500550
1	4.514249	1.710864	-0.377310
1	3.416241	1.780070	0.723688
8	5.588274	-2.073871	-1.478490
1	6.301435	-2.640040	-1.152970
1	5.073595	-2.635785	-2.073690

E(RB3LYP-D3) = -922.0029399 (Hartree)

Sum of electronic and thermal free energies = -921.715427 (Hartree)

AAC2_6H2O_TS1 Cartesian Coordinates

Atomic Number	Coordinates (Angstroms)		
	X	Y	Z
8	0.487582	0.409236	-1.195071
6	1.594379	-0.010241	0.844804
6	2.117190	1.336004	0.252637
1	1.291154	-0.049147	1.887355
1	2.256956	2.154956	0.956719
6	3.425945	0.626434	-0.199955
1	3.432634	0.486458	-1.286295
6	4.736271	1.245767	0.255558
1	4.730475	1.412170	1.337804
1	5.581170	0.590921	0.018825
1	4.909086	2.210002	-0.233489
6	1.136906	1.684337	-0.860411
1	1.606457	2.041090	-1.775825
1	0.359946	2.381116	-0.541048
6	0.431608	-0.337982	-0.062333
8	0.073729	-1.597630	-0.226005
6	3.061343	-1.976162	-0.266630
1	4.112691	-2.208467	-0.464969
1	2.627705	-2.815849	0.283452
1	2.547271	-1.897918	-1.227691
6	2.973462	-0.678079	0.519986
1	3.512067	-0.788573	1.465352
1	-0.659920	-1.703678	-0.911467
8	-0.865253	0.410266	0.788104
1	-1.261329	-0.280948	1.384664
1	-1.602179	0.668624	0.128046
8	-2.826627	1.019559	-0.782759
1	-3.403761	0.260489	-0.567932
1	-3.268112	1.814314	-0.412585
8	-1.916760	-1.979432	-1.884884
1	-2.687211	-1.889426	-1.282282
1	-1.916197	-2.902603	-2.168807
8	-3.838696	-1.443566	0.021666
1	-3.359808	-1.559614	0.866729
1	-4.708312	-1.844962	0.137665
8	-2.066910	-1.539804	2.196756
1	-1.510133	-2.330716	2.197313
1	-2.313764	-1.391666	3.119586
8	-4.006880	3.303394	0.274914
1	-3.474431	3.623789	1.014788
1	-3.986567	4.022967	-0.369624

E(RB3LYP-D3) = -921.9720337 (Hartree)

Sum of electronic and thermal free energies = -921.680945 (Hartree)

AAC2_6H2O_Int Cartesian Coordinates

Atomic Number	Coordinates (Angstroms)		
	X	Y	Z
8	-0.055255	0.200157	-0.008777
6	1.828687	-1.034260	0.691167
6	1.898415	0.419366	1.269162
1	2.051304	-1.855044	1.369818
1	2.251544	0.518679	2.295341
6	2.939496	0.801816	0.179993
1	2.479593	1.440622	-0.582353
6	4.243990	1.418012	0.657396
1	4.701675	0.799996	1.437034
1	4.960354	1.506777	-0.166077
1	4.082264	2.418595	1.072200
6	0.505098	1.000290	1.070620
1	0.499751	2.038693	0.736315
1	-0.124995	0.894312	1.957280
6	0.384118	-1.168410	0.209434
8	0.217608	-1.869648	-0.970675
6	2.890130	-0.918041	-1.798481
1	3.782182	-0.508226	-2.284690
1	2.833800	-1.982700	-2.040780
1	2.015294	-0.426707	-2.228771
6	2.985565	-0.677717	-0.299298
1	3.894405	-1.156255	0.078856
1	-0.740681	-1.880704	-1.213441
8	-0.357786	-1.720304	1.271419
1	-1.428526	0.675603	-0.542828
1	-1.319387	-1.631160	1.096170
8	-3.136448	-1.581699	0.989787
1	-3.542162	-0.704003	1.131505
1	-3.665762	-2.213176	1.492366
8	-2.441493	-1.865597	-1.671292
1	-2.805043	-1.805160	-0.762490
1	-2.579563	-0.983759	-2.040988
8	-2.242729	1.174294	-0.901412
1	-1.939565	2.138992	-1.046386
1	-2.953441	1.167982	-0.185762
8	-1.347106	3.560344	-1.252938
1	-0.627938	3.677524	-0.616043
1	-1.992206	4.247795	-1.037204
8	-4.025727	1.062871	1.005575
1	-3.831185	1.649114	1.749810
1	-4.943217	1.242531	0.757669

E(RB3LYP-D3) = -921.9798128 (Hartree)

Sum of electronic and thermal free energies = -921.682365 (Hartree)

AAC2_6H2O_TS2 Cartesian Coordinates

Atomic Number	Coordinates (Angstroms)		
	X	Y	Z
8	-0.190561	0.425703	-0.436569
6	1.638533	-0.335479	0.976883
6	1.747144	1.185804	0.645308
1	1.634926	-0.617668	2.027340
1	2.104717	1.801054	1.470582
6	2.845685	0.864752	-0.404362
1	2.383789	0.763607	-1.393384
6	4.060761	1.770886	-0.478265
1	4.517912	1.884010	0.510295
1	4.816174	1.360020	-1.155816
1	3.791087	2.767687	-0.842574
6	0.406183	1.645234	0.133981
1	0.461124	2.373789	-0.674639
1	-0.250730	2.006742	0.925788
6	0.319398	-0.824171	0.372634
8	0.429957	-1.830069	-0.525639
6	3.341315	-1.725563	-0.650761
1	4.380524	-1.637509	-0.984695
1	3.243427	-2.673456	-0.113303
1	2.704262	-1.768642	-1.534347
6	3.002257	-0.538919	0.237281
1	3.759032	-0.467187	1.026166
1	-0.445673	-2.115144	-0.905082
8	-0.600149	-1.042502	1.385155
1	-1.309108	0.539853	-0.418100
1	-1.447869	-1.408739	1.038323
8	-3.133262	-1.838422	0.577064
1	-3.403866	-0.948399	0.296390
1	-3.800990	-2.150202	1.200523
8	-1.820333	-2.771479	-1.638973
1	-2.456400	-2.562107	-0.924790
1	-2.092706	-2.225701	-2.387702
8	-2.589078	0.785437	-0.271530
1	-2.864340	1.542750	-0.850565
1	-2.638670	1.088473	0.667095
8	-3.240910	2.807149	-1.929373
1	-2.585069	3.512595	-1.848883
1	-4.085939	3.230995	-1.728382
8	-2.258903	1.097584	2.418624
1	-1.531694	0.463630	2.283828
1	-1.825664	1.917072	2.689666

E(RB3LYP-D3) = -921.9696672 (Hartree)

Sum of electronic and thermal free energies = -921.676877 (Hartree)

AAC2_6H2O_TSuni Cartesian Coordinates

Atomic Number	Coordinates (Angstroms)		
	X	Y	Z
8	-0.321675	-0.358036	-1.282174
6	1.099354	1.160492	0.235789
6	1.753384	0.827331	-1.133922
1	1.028605	2.210312	0.518360
1	2.281067	1.670920	-1.578723
6	2.697390	-0.188247	-0.426882
1	2.334429	-1.208658	-0.586362
6	4.180047	-0.101650	-0.740225
1	4.548310	0.918578	-0.591425
1	4.756736	-0.768709	-0.091606
1	4.380063	-0.385936	-1.778330
6	0.715239	0.254973	-2.088341
1	1.141155	-0.501310	-2.750221
1	0.226899	1.029534	-2.676893
6	-0.275275	0.602714	0.331094
8	-0.970892	0.119916	1.139387
6	1.870017	-0.693333	2.022934
1	2.783053	-1.193232	2.360156
1	1.398072	-0.228346	2.893126
1	1.195445	-1.457583	1.633163
6	2.219064	0.330806	0.958081
1	2.934175	1.055692	1.356209
1	-0.092405	-1.312926	-1.050147
8	-1.225436	1.979299	-0.603275
1	-1.329807	2.650259	0.117233
1	-2.126958	1.584465	-0.764593
8	-3.633021	0.872014	-1.014598
1	-3.513947	-0.087404	-0.834992
1	-3.886482	0.927728	-1.944530
8	-1.060124	-2.845740	1.854803
1	-1.095924	-1.880193	1.915717
1	-1.982120	-3.115702	1.749995
8	-3.222855	-1.812617	-0.446585
1	-2.272127	-1.887821	-0.286847
1	-3.625880	-2.027189	0.404858
8	-1.243915	3.679557	1.521361
1	-1.293812	3.070576	2.270969
1	-0.335394	4.009892	1.539032
8	0.136367	-2.867225	-0.645254
1	-0.417393	-3.414937	-1.217053
1	-0.258684	-2.953856	0.252408

E(RB3LYP-D3) = -921.9562319 (Hartree)

Sum of electronic and thermal free energies = -921.665646 (Hartree)

AAL2_6H2O_TS Cartesian Coordinates

Atomic Number	Coordinates (Angstroms)		
	X	Y	Z
8	-1.303993	-0.259643	-0.547327
6	0.126253	-1.399164	0.931224
6	1.156384	-0.473805	0.215368
1	0.068782	-1.355527	2.018148
1	1.865195	-0.058267	0.930212
6	1.705610	-1.695112	-0.570070
1	1.293273	-1.699298	-1.584275
6	3.211044	-1.886275	-0.605578
1	3.623874	-1.884713	0.408974
1	3.471278	-2.841365	-1.072128
1	3.696820	-1.087996	-1.175150
6	0.505223	0.626265	-0.553612
1	0.481398	0.623853	-1.630456
1	0.067015	1.465580	-0.036152
6	-1.216757	-1.122443	0.349795
8	-2.238285	-1.783686	0.803357
6	0.078436	-3.754385	-0.323414
1	0.758978	-4.480197	-0.778579
1	-0.550356	-4.285058	0.396939
1	-0.566133	-3.358670	-1.114500
6	0.879239	-2.649537	0.341871
1	1.511569	-3.061689	1.131531
1	-3.121578	-1.451499	0.407277
8	2.209692	1.878729	-0.852836
1	1.794782	2.741457	-0.616705
1	2.887129	1.699189	-0.163424
8	0.820374	4.152567	-0.192324
1	-0.044320	3.837692	0.153427
1	0.604612	4.674021	-0.975411
8	-1.555297	3.243489	0.925222
1	-2.050230	3.980265	1.304988
1	-2.201966	2.760021	0.372569
8	-3.323380	1.644392	-0.629553
1	-3.531487	2.038270	-1.486801
1	-2.601264	1.010604	-0.801107
8	-4.494819	-0.882109	-0.093060
1	-5.147526	-0.903206	0.619681
1	-4.319565	0.065939	-0.261319
8	4.141597	1.225738	1.027837
1	4.992659	1.652297	0.862455
1	4.297345	0.289219	0.845777

E(RB3LYP-D3) = -921.9620073 (Hartree)

Sum of electronic and thermal free energies = -921.672335 (Hartree)

AAL1_6H2O_TS1 Cartesian Coordinates

Atomic Number	Coordinates (Angstroms)		
	X	Y	Z
8	-0.010807	0.259072	0.910668
6	-1.587090	-0.187497	-0.851322
6	-2.597792	0.546679	0.025910
1	-1.511666	0.112136	-1.893832
1	-3.275309	1.289686	-0.564276
6	-3.325427	-0.749696	0.429573
1	-3.049432	-1.002211	1.456931
6	-4.828410	-0.822257	0.236658
1	-5.102927	-0.543901	-0.784879
1	-5.181745	-1.839738	0.423997
1	-5.343263	-0.149601	0.928393
6	-2.330962	1.678791	0.786352
1	-2.970462	1.944060	1.626330
1	-1.567889	2.385295	0.470024
6	-0.214002	-0.183288	-0.220831
8	0.704265	-0.723035	-0.984299
6	-1.698499	-2.743577	-0.096011
1	-2.427005	-3.521349	0.149395
1	-1.019010	-3.140112	-0.854661
1	-1.119208	-2.529157	0.807331
6	-2.422580	-1.507814	-0.598213
1	-2.989007	-1.733219	-1.503634
1	1.627618	-0.740256	-0.546528
8	3.091671	-0.743444	-0.001880
1	3.601554	-1.567477	-0.169807
1	3.004690	-0.639447	0.969382
8	1.120830	2.881651	0.635221
1	1.005658	3.584022	1.287274
1	0.740459	2.081510	1.031480
8	4.473282	-3.070417	-0.539302
1	4.949694	-3.002223	-1.377181
1	5.159238	-3.239129	0.119965
8	3.429967	1.974594	-0.768203
1	2.678529	2.347937	-0.273468
1	3.397639	1.022590	-0.562819
8	2.267077	-0.085609	2.551577
1	2.603551	0.803846	2.719799
1	1.435941	0.054749	2.058850
8	-0.187224	2.874841	-1.873605
1	0.263476	2.895621	-1.007353
1	0.004977	1.997090	-2.224834

E(RB3LYP-D3) = -921.9359421 (Hartree)

Sum of electronic and thermal free energies = -921.656348 (Hartree)

AAL1_6H2O_Int Cartesian Coordinates

Atomic Number	Coordinates (Angstroms)		
	X	Y	Z
8	0.079058	0.062423	1.064645
6	-1.515372	-0.273582	-0.695868
6	-2.580378	0.446836	0.054909
1	-1.466363	0.120950	-1.717552
1	-3.199352	2.207421	-0.682091
6	-3.330760	-0.761004	0.352476
1	-2.921567	-0.910566	1.386296
6	-4.844281	-0.843360	0.377203
1	-5.235027	-0.675054	-0.628565
1	-5.154296	-1.833133	0.715380
1	-5.264591	-0.093500	1.050431
6	-2.808637	1.844893	0.290639
1	-3.573674	2.029115	1.044234
1	-1.877460	2.392122	0.457108
6	-0.126577	-0.270443	-0.097946
8	0.783133	-0.690551	-0.940591
6	-1.751143	-2.844983	-0.013295
1	-2.518420	-3.604291	0.159042
1	-1.016943	-3.255263	-0.711091
1	-1.250498	-2.643958	0.938373
6	-2.384259	-1.590693	-0.579020
1	-2.865708	-1.783444	-1.537980
1	1.726265	-0.702219	-0.537464
8	3.198454	-0.655066	-0.060727
1	3.723472	-1.472194	-0.216860
1	3.159018	-0.506844	0.907814
8	0.778948	2.852377	0.629397
1	0.534290	3.628198	1.149105
1	0.430923	2.086661	1.110685
8	4.614260	-2.970820	-0.542054
1	5.089896	-2.923010	-1.381790
1	5.303238	-3.102888	0.122490
8	3.282661	2.172937	-0.580370
1	2.455853	2.421460	-0.130104
1	3.323793	1.204991	-0.496442
8	2.511813	0.195931	2.473664
1	2.779844	1.123642	2.450441
1	1.634990	0.184074	2.042888
8	-0.037791	2.484410	-2.070168
1	0.203927	2.624860	-1.134928
1	0.342538	1.623768	-2.284376

E(RB3LYP-D3) = -921.9630137 (Hartree)

Sum of electronic and thermal free energies = -921.687624 (Hartree)

AAL1_6H2O_TS2 Cartesian Coordinates

Atomic Number	Coordinates (Angstroms)		
	X	Y	Z
8	-0.517436	0.134639	0.432624
6	1.156912	0.942427	-1.093631
6	1.946110	-0.352685	-0.934486
1	1.087828	1.323736	-2.109919
1	2.550177	-0.607721	-2.001602
6	2.870440	0.234269	0.120005
1	2.505103	-0.115101	1.091105
6	4.368410	0.016517	0.002386
1	4.730890	0.346823	-0.975080
1	4.888869	0.586849	0.775664
1	4.620787	-1.039581	0.128792
6	1.753662	-1.631311	-1.426622
1	2.477134	-2.403961	-1.199553
1	0.990550	-1.818801	-2.172091
6	-0.207486	0.916533	-0.466401
8	-1.015549	1.836544	-0.941585
6	1.841785	2.505767	0.969838
1	2.721174	2.813631	1.542318
1	1.328620	3.407680	0.625309
1	1.173794	1.963816	1.645356
6	2.270555	1.639956	-0.198712
1	2.934217	2.191675	-0.867284
1	-1.919138	1.821041	-0.486603
8	-3.418438	1.779229	0.116103
1	-3.818546	2.636557	0.310370
1	-3.340216	1.300417	0.972013
8	-2.245905	-2.010179	-0.382830
1	-2.778299	-2.327534	0.358916
1	-1.784813	-1.227548	-0.034847
8	0.380851	-3.204645	-0.107933
1	-0.529708	-2.920659	-0.308721
1	0.556171	-2.801510	0.765546
8	-4.010721	-0.238813	-1.831201
1	-3.450431	-0.925444	-1.426658
1	-3.912264	0.517152	-1.226015
8	-2.697489	0.219084	2.271052
1	-3.081902	-0.656037	2.128665
1	-1.855543	0.191118	1.782067
8	0.715144	-1.656625	2.236590
1	0.396204	-0.937631	1.660679
1	-0.016631	-1.821681	2.844753

E(RB3LYP-D3) = -921.9463717 (Hartree)

Sum of electronic and thermal free energies = -921.662477 (Hartree)

REFERENCES

(1) Gómez-Bombarelli, R.; Calle, E.; Casado, J. Mechanisms of Lactone Hydrolysis in Neutral and Alkaline Conditions. *J. Org. Chem.* **2013**, *78*, 6868-6879.

(2) Gómez-Bombarelli, R.; Calle, E.; Casado, J. Mechanisms of Lactone Hydrolysis in Acidic Conditions. *J. Org. Chem.* **2013**, *78*, 6880-6889.
